# Optimal assembly for high throughput shotgun sequencing

**DOI:** 10.1186/1471-2105-14-S5-S18

**Published:** 2013-07-09

**Authors:** Guy Bresler, Ma'ayan Bresler, David Tse

**Affiliations:** 1 Department of EECS, UC Berkeley, California, USA

## Abstract

We present a framework for the design of optimal assembly algorithms for shotgun sequencing under the criterion of complete reconstruction. We derive a lower bound on the read length and the coverage depth required for reconstruction in terms of the repeat statistics of the genome. Building on earlier works, we design a de Brujin graph based assembly algorithm which can achieve very close to the lower bound for repeat statistics of a wide range of sequenced genomes, including the GAGE datasets. The results are based on a set of necessary and sufficient conditions on the DNA sequence and the reads for reconstruction. The conditions can be viewed as the shotgun sequencing analogue of Ukkonen-Pevzner's necessary and sufficient conditions for Sequencing by Hybridization.

## Introduction

### Problem statement

DNA sequencing is the basic workhorse of modern day biology and medicine. Since the sequencing of the Human Reference Genome ten years ago, there has been an explosive advance in sequencing technology, resulting in several orders of magnitude increase in throughput and decrease in cost. Multiple "next-generation" sequencing platforms have emerged. All of them are based on the whole-genome shotgun sequencing method, which entails two steps. First, many short reads are extracted from random locations on the DNA sequence, with the length, number, and error rates of the reads depending on the particular sequencing platform. Second, the reads are assembled to reconstruct the original DNA sequence.

Assembly of the reads is a major algorithmic challenge, and over the years dozens of assembly algorithms have been proposed to solve this problem [1]. Nevertheless, the assembly problem is far from solved, and it is not clear how to compare algorithms nor where improvement might be possible. The difficulty of comparing algorithms is evidenced by the recent assembly evaluations Assemblathon 1 [2] and GAGE [3], where which assembler is "best" depends on the particular dataset as well as the performance metric used. In part this is a consequence of metrics for partial assemblies: there is an inherent tradeoff between larger contiguous fragments (contigs) and fewer mistakes in merging contigs (misjoins). But more fundamentally, independent of the metric, performance depends critically on the dataset, i.e. length, number, and quality of the reads, as well as the complexity of the genome sequence.

With an eye towards the near future, we seek to understand the interplay between these factors by using the intuitive and unambiguous metric of *complete *reconstruction. The notion of complete reconstruction can be thought of as a mathematical idealization of the notion of "finishing" a sequencing project as defined by the National Human Genome Research Institute [4], where finishing a chromosome requires at least 95% of the chromosome to be represented by a contiguous sequence. Note that this objective of reconstructing the original DNA sequence from the reads contrasts with the many *optimization-based *formulations of assembly, such as shortest common superstring (SCS) [5], maximum-likelihood [6], [7], and various graph-based formulations [8], [9]. When solving one of these alternative formulations, there is no guarantee that the optimal solution is indeed the original sequence.

Given the goal of complete reconstruction, the most basic questions are 1) **feasibility**: given a set of reads, is it *possible *to reconstruct the original sequence? 2) **optimality**: which *algorithms *can successfully reconstruct whenever it is feasible to reconstruct? The feasibility question is a measure of the intrinsic *information *each read provides about the DNA sequence, and for given sequence statistics depends on characteristics of the sequencing technology such as read length and noise statistics. As such, it can provide an algorithm-independent basis for evaluating the efficiency of a sequencing technology. Equally important, algorithms can be evaluated on their relative read length and data requirements, and compared against the fundamental limit.

In studying these questions, we consider the most basic shotgun sequencing model where *N *noiseless reads (i.e. exact subsequences) of a fixed length *L *base pairs are uniformly and independently drawn from a DNA sequence of length *G*. In this statistical model, feasibility is rephrased as the question of whether, for given sequence statistics, the correct sequence can be reconstructed with probability 1 *- ∈ *when *N *reads of length *L *are sampled from the genome. We note that answering the feasibility question of whether each *N, L *pair is sufficient to reconstruct is equivalent to finding the minimum required *N *(or the *coverage depth c *= *NL*/*G*) as a function of *L*.

A lower bound on the minimum coverage depth needed was obtained by Lander and Waterman [10]. Their lower bound *c*_LW _= *c*_LW_(*L, ∈*) is the minimum number of randomly located reads needed to cover the entire DNA sequence with a given target success probability 1 - *∈*. While this is clearly a necessary condition, it is in general not tight: only requiring the reads to cover the entire genome sequence does not guarantee that consecutive reads can actually be stitched back together to recover the original sequence. Characterizing when the reads can be reliably stitched together, i.e. determining feasibility, is an open problem. In fact, the ability to reconstruct depends crucially on the *repeat statistics *of the DNA sequence.

An earlier work [11] has answered the feasibility and optimality questions under an i.i.d. model for the DNA sequence. However, real DNA, especially those of eukaryotes, have much longer and complex repeat structures. Here, we are interested in determining feasibility and optimality given *arbitrary *repeat statistics. This allows us to evaluate algorithms on statistics from already sequenced genomes, and gives confidence in predicting whether the algorithms will be useful for an *unseen *genome with similar statistics.

### Results

**Figure 1:**
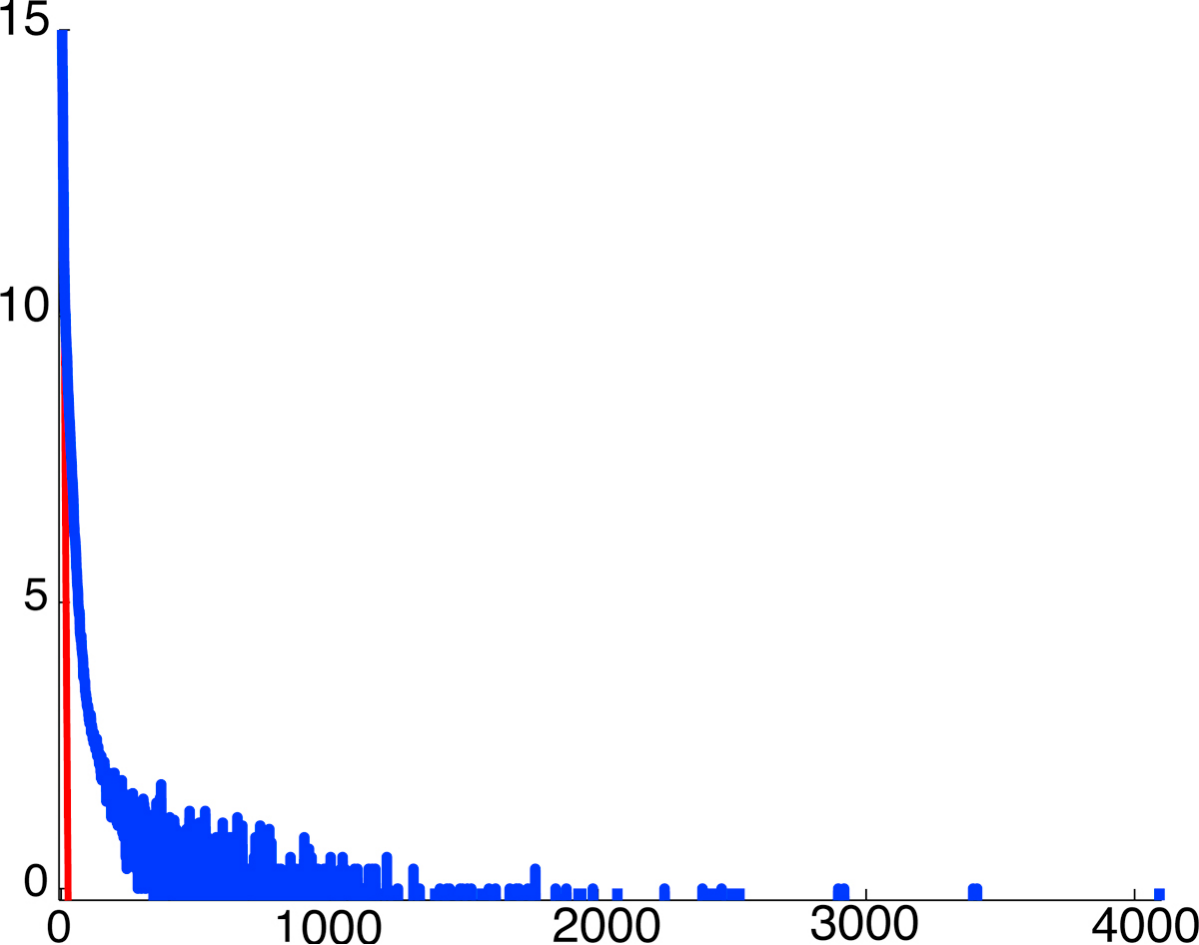
**For hc19, a log plot of number of repeats as a function of the repeat length *ℓ***. Red line is what would have been predicted by an i.i.d. fit.

**Figure 2:**
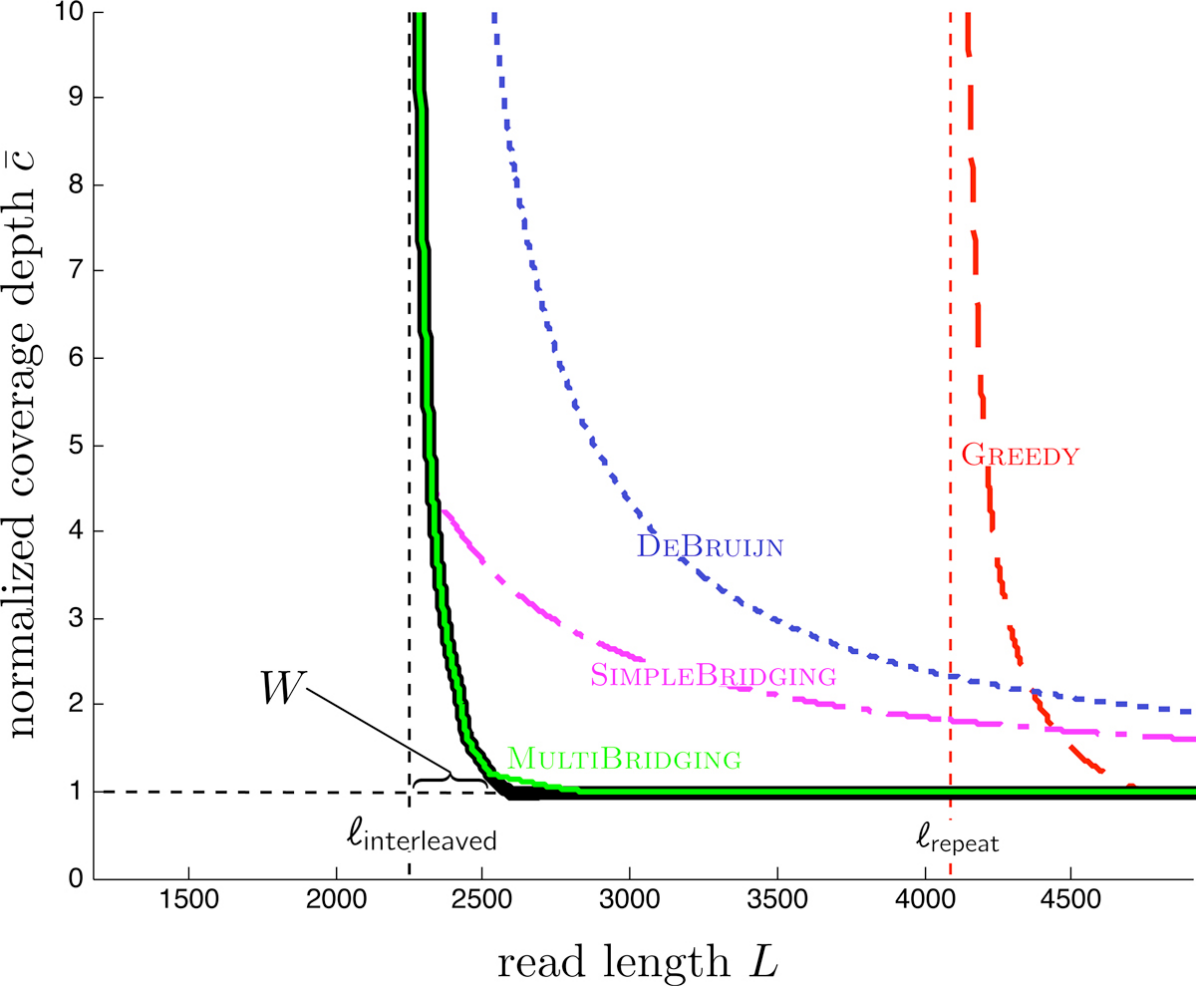
**Thick black lines are lower bounds on feasibility which holds for all algorithms, and colored curves are performance achieved by specific algorithms**. Four such curves are shown: the greedy algorithm and three de Brujin graph based algorithms.

Our approach results in a pipeline, which takes as input a genome sequence and desired success probability 1 - *∈*, computes a few simple repeat statistics, and from these statistics computes a feasibility plot that indicates for which *L*, *N *reconstruction is possible. Figure 1 displays the simplest of the statistics, the number of repeats as a function of the repeat length *ℓ*. Figure 2 shows the resulting feasibility plot produced for the statistics of human chromosome 19 (henceforth hc19) with success probability 99%. The horizontal axis signifies read length *L *and the vertical axis signifies the normalized coverage depth $\bar{c}:=c/c_{LW}$, the coverage depth *c *normalized by *c*_LW_, the coverage depth required as per Lander-Waterman [10] in order to cover the sequence.

Since the coverage depth must satisfy *c *≥ *c*_LW_, the normalized coverage depth satisfies $\bar{c}\geq 1$, and we plot the horizontal line $\bar{c}=1$. This lower bound holds for *any *assembly algorithm. In addition, there is another lower bound, shown as the thick black nearly vertical line in Figure 2. In contrast to the coverage lower bound, this lower bound is a function of the repeat statistics. It has a vertical asymptote at *L*_crit _:= max{*ℓ*_interleaved_, *ℓ*_triple_} + 1, where *ℓ*_interleaved _is the length of the longest interleaved repeats in the DNA sequence and *ℓ*_triple _is the length of the longest triple repeat (see Section for precise definitions). Our lower bound can be viewed as a generalization of a result of Ukkonen [12] for Sequencing by Hybridization to the shotgun sequencing setting.

Each colored curve in the feasibility plot is the lower boundary of the set of feasible *N,L *pairs for a specific algorithm. The rightmost curve is the one achieved by the greedy algorithm, which merges reads with largest overlaps first (used for example in TIGR [13], CAP3 [14], and more recently SSAKE [15]). As seen in Figure 2, its performance curve asymptotes at *L *= *ℓ*_repeat_, the length of the longest repeat. De Brujin graph based algorithms (e.g. [16] and [8]) take a more global view via the construction of a de Brujin graph out of all the K-mers of the reads. The performance curves of all K-mer graph based algorithms asymptote at read length *L *= *L*_crit_, but different algorithms use read information in a variety of ways to resolve repeats in the K-mer graph and thus have different coverage depth requirement beyond read length *L*_crit_. By combining the ideas from several existing algorithms (including [8], [17]) we designed MULTIBRIDGING, which is very close to the lower bound for this dataset. Thus Figure 2 answers, up to a very small gap, the feasibility of assembly for the repeat statistics of hc19, where successful reconstruction is desired with probability 99%.

**Figure 3:**
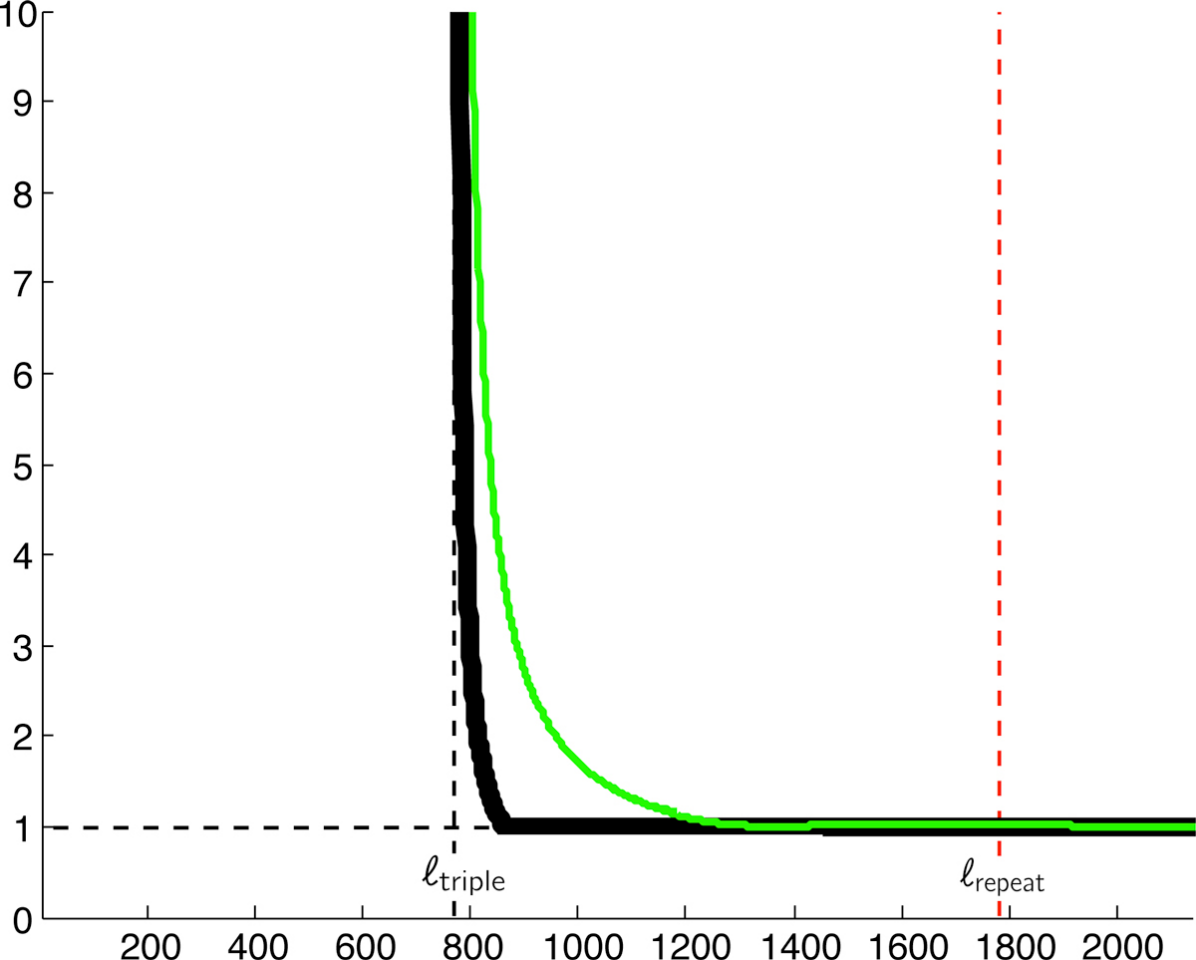
**Performance of MULTIBRIDGING on *P Marinus*, where *ℓ*_triple _*> ℓ*_interleaved_**.

We produce similar plots for a dozen or so datasets (Additional file 1). For datasets where *ℓ*_interleaved _is significantly larger than *ℓ*_triple _(the majority of the datasets we looked at, including those used in the recent GAGE assembly algorithm evaluation [3]), MULTIBRIDGING is near optimal, thus allowing us to characterize the fundamental limits for these repeat statistics (Figure 9). On the other hand, if *ℓ*_triple _is close to or larger than *ℓ*_interleaved_, there is a gap between the performance of MULTIBRIDGING and the lower bound (see for example Figure 3). The reason for the gap is explained later in the paper.

An interesting feature of the feasibility plots is that for typical repeat statistics exhibited by DNA data, the minimum coverage depth is characterized by a *critical phenomenon*: If the read length *L *is below *L*_crit _= *ℓ*_interleaved_, reliable reconstruction of the DNA sequence is impossible no matter what the coverage depth is, but if the read length *L *is slightly above *L*_crit_, then covering the sequence suffices, i.e. $\bar{c}=c/c_{\text{LW}}=1$. The sharpness of the critical phenomenon is described by the size of the *critical window*, which refers to the range of *L *over which the transition from one regime to the other occurs. For the case when MULTIBRIDGING is near optimal, the width *W *of the window size can be well approximated as:

(1) $$W\approx \frac{L_{\text{crit}}}{2r+1},\;\;where\;\;r:=\frac{\text{log}\frac{G}{L_{\text{crit}}}}{\text{log}\varepsilon^{-1}}.$$

For the hc19 dataset, the critical window size evaluates to about 19% of *L*_crit_.

In Sections and, we discuss the underlying analysis and algorithm design supporting the plots. The curves are all computed from formulas, which are validated by simulations. We return in Section to put our contributions in a broader perspective and discuss extensions to the basic framework. All proofs can be found in the appendix.

## Lower bounds

In this section we discuss lower bounds, due to coverage analysis and certain repeat patterns, on the required coverage depth and read length. The style of analysis here is continued in Section, in which we search for an assembly algorithm that performs close to the lower bounds.

### Coverage bound

Lander and Waterman's coverage analysis [10] gives the well known condition for the number of reads *N*_LW _required to cover the entire DNA sequence with probability at least 1 - *∈*. In the regime when *L *≪ *G*, one may make the standard assumption that the starting locations of the *N *reads follow a Poisson process with rate λ = *N/G*, and the number *N*_LW _is to a very good approximation given by the solution to the equation

(2) $$N_{\text{LW}}=\frac{G}{L}\text{log}\frac{N_{\text{LW}}}{\varepsilon }.$$

The corresponding coverage depth is *c*_LW _= *N*_LW_*L*/*G*. This is our baseline coverage depth against which to compare the coverage depth of various algorithms. For each algorithm, we will plot

$$\bar{c}:=\frac{c}{c_{\text{LW}}}=\frac{N}{N_{\text{LW}}},$$

the coverage depth required by that algorithm normalized by *c*_LW_. Note that $\bar{c}$ is also the ratio of the number of reads *N *required by an algorithm to *N*_LW_. The requirement $\bar{c}\geq 1$ is due to the lower bound on the number of reads obtained by the Lander-Waterman coverage condition.

### Ukkonen's condition

**Figure 4:**
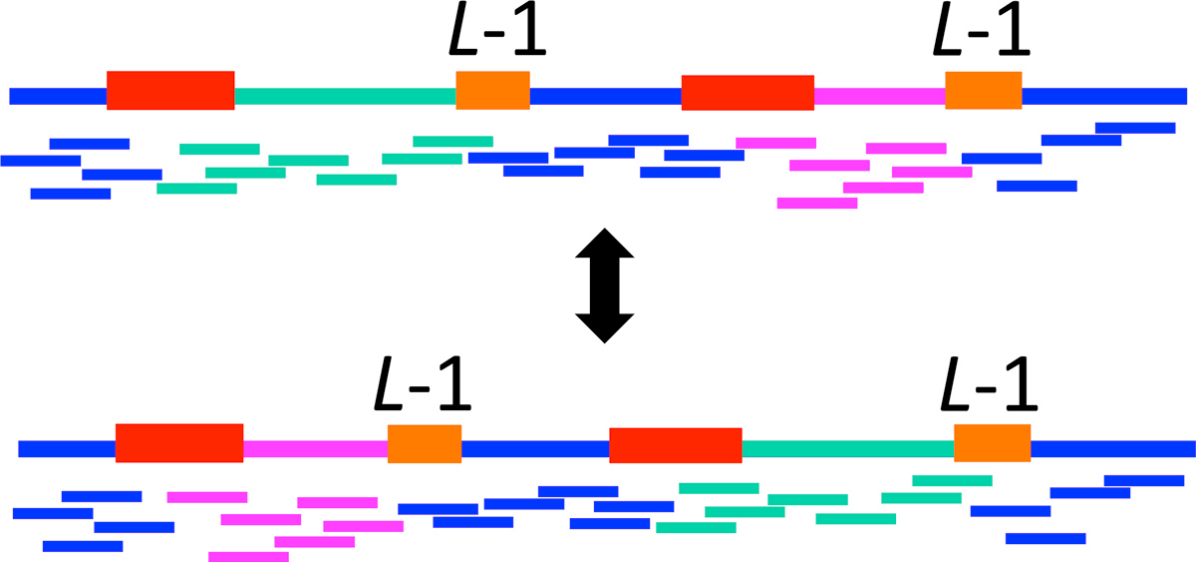
**The likelihood of observing the reads under two possible sequences (the green and magenta segments swapped) is the same**. Here, the two red subsequences form a repeat and the two orange subsequences form another repeat.

A second constraint on reads arises from repeats. A lower bound on the read length *L *follows from Ukkonen's condition [12]: if there are *interleaved repeats *or *triple repeats *in the sequence of length at least *L *- 1, then the likelihood of observing the reads is the same for more than one possible DNA sequence and hence correct reconstruction is not possible. Figure 4 shows an example with interleaved repeats. (Note that we assume 1-*∈ >*1/2, so random guessing between equally likely sequences is not viable.)

We take a moment to carefully define the various types of repeats. Let ${\text{s}}_{t}^{\ell }$ denote the length-*ℓ* subsequence of the DNA sequence **s **starting at position *t*. A *repeat *of length *ℓ* is a subsequence appearing twice, at some positions *t*_1_*, t*_2 _(so ${\text{s}}_{t_{1}}^{\ell }={\text{s}}_{t_{2}}^{\ell }$) that is maximal (i.e. *s*(*t*_1 _- 1) ≠ *s*(*t*_2 _- 1) and *s*(*t*_1 _+ *ℓ*) ≠ *s*(*t*_2 _+ *ℓ*)). Similarly, a *triple repeat *of length *ℓ* is a subsequence appearing three times, at positions *t*_1_*, t*_2_*, t*_3_, such that ${\text{s}}_{t_{1}}^{\ell }={\text{s}}_{t_{2}}^{\ell }={\text{s}}_{t_{3}}^{\ell }$, and such that neither of *s*(*t*_1 _- 1) = *s*(*t*_2 _- 1) = *s*(*t*_3 _- 1) nor *s*(*t*_1 _+ *ℓ*) = *s*(*t*_2 _+ *ℓ*) = *s*(*t*_3 _+ *ℓ*) holds. (Note that a subsequence that is repeated *f *times gives rise to ${(}_{2}^{f})$ repeats and ${(}_{3}^{f})$ triple repeats.) A *copy *is a single one of the instances of the subsequence's appearances. A *pair *of repeats refers to two repeats, each having two copies. A pair of repeats, one at positions *t*_1_*, t*_3 _with *t*_1 _*< t*_3 _and the second at positions *t*_2_*, t*_4 _with *t*_2 _*< t*_4_, is *interleaved *if *t*_1 _*< t*_2 _*< t*_3 _*< t*_4 _or *t*_2 _*< t*_1 _*< t*_4 _*< t*_3 _(Figure 4). The length of a pair of interleaved repeats is defined to be the length of the shorter of the two repeats.

Ukkonen's condition implies a lower bound on the read length,

$$L>L_{\text{crit}}:=\text{max}\left\{\ell_{\text{interleaved}\;},\;\ell_{\text{triple}}\right\}+1.$$

Here *ℓ*_interleaved _is the length of the longest pair of interleaved repeats on the DNA sequence and *ℓ*_triple _is the length of the longest triple repeat.

**Figure 5:**
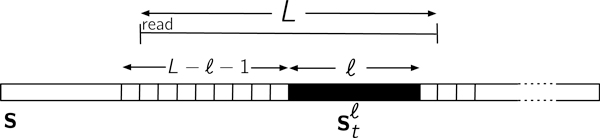
**A subsequence **${\text{s}}_{t}^{\ell }$**is bridged if and only if there exists at least one read which covers at least one base on both sides of the subsequence, i.e. the read arrives in the preceding length *L*-*ℓ*-1 interval**.

Ukkonen's condition says that for read lengths less than *L*_crit_, reconstruction is impossible no matter what the coverage depth is. But it can be generalized to provide a lower bound on the coverage depth for read lengths greater than *L*_crit_, through the important concept of *bridging *as shown in Figure 5. We observe that in Ukkonen's interleaved or triple repeats, the actual length of the repeated subsequences is irrelevant; rather, to cause confusion it is enough that all the copies of the pertinent repeats are unbridged. This leads to the following theorem.

**Theorem 1**. *Given a DNA sequence ***s ***and a set of reads, if there is a pair of interleaved repeats or a triple repeat whose copies are all unbridged, then there is another sequence ***s' ***of the same length under which the likelihood of observing the reads is the same*.

For brevity, we will call a repeat or a triple repeat *bridged *if at least one copy of the repeat is bridged, and a pair of interleaved repeats *bridged *if at least one of the repeats is bridged. Thus, the above theorem says that a necessary condition for reconstruction is that all interleaved and triple repeats are bridged.

How does Theorem 1 imply a lower bound on the coverage depth? Focus on the longest pair of interleaved repeats and suppose the read length *L *is between the lengths of the shorter and the longer repeats. The probability this pair is unbridged is ${\left(p_{\ell_{\text{inter}1\text{eaved}}}^{\text{unbridged}}\right)}^{2}$, where

(3) $$\begin{array}{llll} p_{\ell }^{\text{unbridged}} & :=\mathbb{P}\left[\ell -\text{length}\;\;\text{subseq}\text{.}\;\;\;\text{is}\;\;\text{unbridged}\right]\; & & \;\\ & =e^{\frac{N}{G}{\left(L-\ell -1\right)}^{+}}.\; & & \;\\ & \; & \end{array}$$

Theorem 1 implies that the probability of making an error in the reconstruction is at least 1/2 if this event occurs. Hence, the requirement that *P*_error _≤ *∈ *implies a lower bound on the number of reads *N*:

(4) $$N\geq \frac{G}{\left(L-\ell_{\text{interleaved}}-1\right)\text{ln}\left(1/\left(2\varepsilon \right)\right)}.$$

A similar lower bound can be derived using the longest triple repeat. A slightly tighter lower bound can be obtained by taking into consideration the bridging of *all *the interleaved and triple repeats, not only the longest one, resulting in the black curve in Figure 2.

## Towards optimal assembly

We now begin our search for algorithms performing close to the lower bounds derived in the previous section. Algorithm assessment begins with obtaining deterministic sufficient conditions for success in terms of repeat-bridging. We then find the necessary *N *and *L *in order to satisfy these sufficient conditions with a target probability 1 *- ∈*. The required coverage depth for each algorithm depends only on certain repeat statistics extracted from the DNA data, which may be thought of as *sufficient statistics*.

### Greedy algorithm

The greedy algorithm, denoted GREEDY, with pseudocode in the supplementary material, is described as follows. Starting with the initial set of reads, the two fragments (i.e. subsequences) with maximum length overlap are merged, and this operation is repeated until a single fragment remains. Here the overlap of two fragments **x**, **y **is a suffix of **x **equal to a prefix of **y**, and merging two fragments results in a single longer fragment.

**Theorem 2**. GREEDY*reconstructs the original sequence ***s ***if every repeat is bridged*.

Theorem 2 allows us to determine the coverage depth required by GREEDY: we must ensure that all repeats are bridged. By the union bound,

$$\mathbb{P}\left[\text{some}\;\;\text{repeat}\;\;\text{is}\;\;\text{unbridged}\right]\leq \underset{m}{\sum }a_{m}{\left(p_{m}^{\text{unbridged}}\right)}^{2},$$

where $p_{m}^{\text{unbridged}}$ is defined in (3) and *a_m _*is the number of repeats of length *m*. Setting the right-hand side of (5) to ∈ ensures *P*_error _*≤ ∈ *and yields the performance curve of GREEDY in Figure 2. Note that the repeat statistics {*a_m_*} are sufficient to compute this curve.

GREEDY requires *L > ℓ*_repeat _+ 1, whereas the lower bound has its asymptote at *L *= *ℓ*_interleaved _+ 1. In chromosome 19, for instance, there is a large difference between *ℓ*_interleaved _= 2248 and *ℓ*_repeat _= 4092, and in Figure 2 we see a correspondingly large gap. GREEDY is evidently sub-optimal in handling interleaved repeats. Its strength, however, is that once the reads are slightly longer than *ℓ*_repeat_, coverage of the sequence is sufficient for correct reconstruction. Thus if *ℓ*_repeat _*≈ ℓ*_interleaved_, then GREEDY is close to optimal.

### *K*-mer algorithms

The greedy algorithm fails when there are unbridged repeats, even if there are no unbridged *interleaved *repeats, and therefore requires a read length much longer than that required by Ukkonen's condition. As we will see, *K*-mer algorithms do not have this limitation.

### Background

In the introduction we mention Sequencing By Hybridization (SBH), for which Ukkonen's condition was originally introduced. In the SBH setting, an optimal algorithm matching Ukkonen's condition is known, due to Pevzner [18].

Pevzner's algorithm is based on finding an appropriate cycle in a *K*-mer graph (also known as a de Bruijn graph) with *K *= *L *- 1 (see e.g. [19] for an overview). A *K*-mer graph is formed by first creating a node in the graph for each unique *K*-mer (length *K *subsequence) in the set of reads, and then adding an edge with overlap *K *- 1 between any two nodes representing *K-*mers that are *adjacent *in a read, i.e. offset by a single nucleotide. Edges thus correspond to unique (*K *+ 1)-mers in **s **and paths correspond to longer subsequences obtained by merging the constituent nodes. There exists a cycle corresponding to the original sequence **s**, and reconstruction entails finding this cycle.

**Figure 6:**
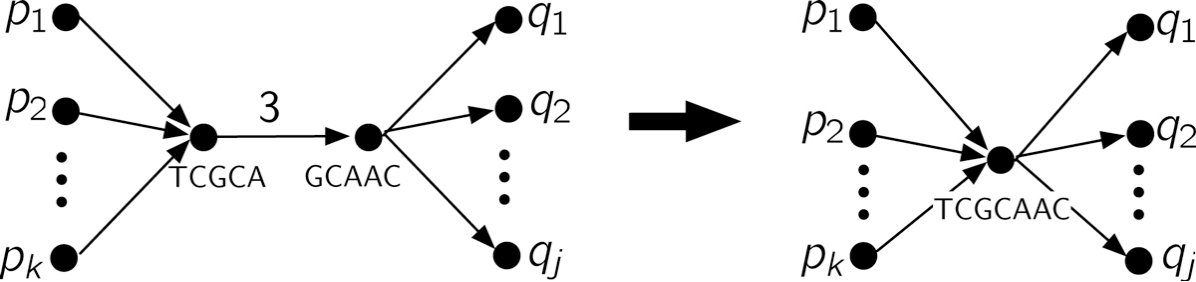
**Contracting an edge by merging the incident nodes**. Repeating this operation results in the condensed graph.

As is common, we will replace edges corresponding to an unambiguous path by a single node (c.f. Figure 6). Since the subsequences at some nodes are now longer than *K*, this is no longer a *K*-mer graph, and we call the more general graph a sequence graph. The simplified graph is called the *condensed sequence graph*.

The condensed graph has the useful property that if the original sequence **s **is reconstructible, then **s **is determined by a unique Eulerian cycle:

**Theorem 3**. *Let $G_{0}$ be the K-mer graph constructed from the *(*K *+ 1)*-spectrum $S_{K+1}$ of ***s***, and let *G *be the condensed sequence graph obtained from *$G_{0}$. *If Ukkonen's condition is satisfied, i.e. there are no triple or interleaved repeats of length at least K, then there is a unique Eulerian cycle *C *in *G *and *C *corresponds to ***s**.

Theorem 3 characterizes, deterministically, the values of *K *for which reconstruction from the (*K *+ 1)-spectrum is possible. We proceed with application of the *K*-mer graph approach to shotgun sequencing data.

### Basic *K*-mer algorithm

Starting with Idury and Waterman [16], and then Pevzner et al.'s [8] EULER algorithm, most current assembly algorithms for shotgun sequencing are based on the *K*-mer graph. Idury and Waterman [16] made the key observation that SBH with subsequences of length *K*+1 can be *emulated *by shotgun sequencing if each read overlaps the subsequent read by *K*: the set of all (*K *+1)-mers within the reads is equal to the (*K*+1)-spectrum $S_{K+1}$. The resultant algorithm DEBRUIJN which consists of constructing the *K-*mer graph from the (*K*+1)-spectrum observed in the reads, condensing the graph, and then identifying an Eulerian cycle, has sufficient conditions for correct reconstruction as follows.

**Theorem 4**. DEBRUIJN*with parameter choice K reconstructs the original sequence ***s ***if:*

(a) K > ℓ_interleaved_

(b) K > ℓ_triple_

(c) adjacent reads overlap by at least K

Lander and Waterman's coverage analysis applies also to Condition (c) of Theorem 4, yielding a normalized coverage depth requirement $\bar{c}=1/\left(1-K/L\right)$. The larger the overlap *K*, the higher the coverage depth required. Conditions (a) and (b) say that the smallest *K *one can choose is *K *= max{*ℓ*_triple_, *ℓ*_interleaved_} + 1, so

(6) $$\bar{c}=\frac{1}{1-\frac{\text{max}\left\{\ell_{\text{trip}1\text{e}},\ell_{\text{interleaved}}\right\}+1}{L}}\;\;.$$

The performance of DEBRUIJN is plotted in Figure 2. DEBRUIJN significantly improves on GREEDY by obtaining the correct first order performance: given sufficiently many reads, the read length *L *may be decreased to {*ℓ*_triple_, *ℓ*_interleaved_} + 1. Still, the number of reads required to approach this critical length is far above the lower bound. The following subsection pursues reducing *K *in order to reduce the required number of reads.

### Improved *K*-mer algorithms

Algorithm DEBRUIJN ignores a lot of information contained in the reads, and indeed all of the *K*-mer based algorithms proposed by the sequencing community (including [16], [8], [20], [21], [22], [23]) use the read information to a greater extent than the naive DEBRUIJN algorithm. Better use of the read information, as described below in algorithms SIMPLEBRIDGING and MULTIBRIDGING, will allow us to relax the condition *K >*max{*ℓ*_interleaved_, *ℓ*_triple_} for success of DEBRUIJN, which in turn reduces the high coverage depth required by Condition (c).

Existing algorithms use read information in a variety of distinct ways to resolve repeats. For instance, Pevzner et al. [8] observe that for graphs where each edge has multiplicity one, if one copy of a repeat is bridged, the repeat can be resolved through what they call a "detachment". The algorithm SIMPLEBRIDGING described below is very similar, and resolves repeats with two copies if at least one copy is bridged.

Meanwhile, other algorithms are better suited to higher edge multiplicities due to higher order repeats; IDBA (Iterative DeBruijn Assembler) [17] creates a series of *K*-mer graphs, each with larger *K*, and at each step uses not just the reads to identify adjacent *K*-mers, but also all the unbridged paths in the *K*-mer graph with smaller *K*. Although not stated explicitly in their paper, we observe here that if all copies of every repeat are bridged, then IDBA correctly reconstructs.

However, it is suboptimal to require that *all *copies of every repeat up to the maximal *K *be bridged. We introduce MULTIBRIDGING, which combines the aforementioned ideas to simultaneously allow for single-bridged double repeats, triple repeats in which all copies are bridged, and unbridged non-interleaved repeats.

## SimpleBridging

SIMPLEBRIDGING improves on DEBRUIJN by resolving bridged 2-repeats (i.e. a repeat with exactly two copies in which at least one copy is bridged by a read). Condition (a) *K > ℓ*_interleaved _for success of DEBRUIJN (ensuring that no interleaved repeats appear in the initial *K*-mer graph) is updated to require only no *unbridged *interleaved repeats, which matches the lower bound. With this change, Condition (b) *K > ℓ*_triple _forms the bottleneck for typical DNA sequences. Thus SIMPLEBRIDGING is optimal with respect to interleaved repeats, but it is suboptimal with respect to triple repeats.

**Figure 7:**
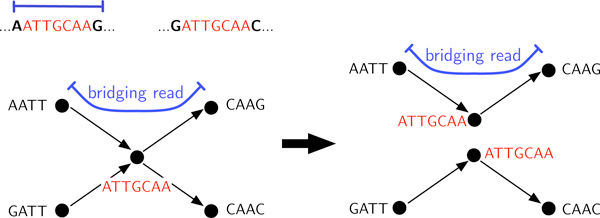
**An example of the bridging step in SIMPLEBRIDGING**.

SIMPLEBRIDGING deals with repeats by performing surgery on certain nodes in the sequence graph. In the sequence graph, a repeat corresponds to a node we call an *X-node*, a node with in-degree and out-degree each at least two (e.g. Figure 7). A self-loop adds one each to the in degree and out-degree. The cycle C(s) traverses each X-node at least twice, so X-nodes correspond to repeats in **s**. We call an X-node traversed exactly twice a 2-X-node; these nodes correspond to 2-repeats, and are said to be bridged if the corresponding repeat in **s **is bridged.

In the repeat resolution step of SIMPLEBRIDGING (illustrated in Figure 7), bridged 2-X-nodes are duplicated in the graph and incoming and outgoing edges are inferred using the bridging read, reducing possible ambiguity.

**Theorem 5**. SIMPLEBRIDGING*with parameter choice K reconstructs the original sequence ***s ***if:*

(a) all interleaved repeats are bridged

(b) K > ℓ_triple_

*(c) adjacent reads overlap by at least K*.

By the union bound,

(7) $$\begin{array}{c} \mathbb{P}\left[\text{some}\;\;\text{interleaved}\;\;\text{repeat}\;\;\text{is}\;\;\text{unbridged}\right]\\ \leq \underset{m,n}{\sum }b_{m,n}{\left(p_{m}^{\text{unbridged}}\right)}^{2}{\left(p_{n}^{\text{unbridged}}\right)}^{2} \end{array}$$

where *b_m,n _*is the number of interleaved repeats in which one repeat is of length *m *and the other is of length *n*. To ensure that condition (a) in the above theorem fails with probability no more than *∈*, the right hand side of (7) is set to be *∈*; this imposes a constraint on the coverage depth. Furthermore, conditions (b) and (c) imply that the normalized coverage depth $\bar{c}\geq 1/\left(1-\left(\ell_{\text{triple}}+1\right)/L\right)$. These two constraints together yield the performance curve of SIMPLEBRIDGING in Figure 2.

## MultiBridging

We now turn to triple repeats. As previously observed, it can be challenging to resolve repeats with more than one copy [8], because an edge into the repeat may be paired with more than one outgoing edge. As discussed above, our approach here shares elements with IDBA [17]: we note that increasing the node length serves to resolve repeats. Unlike IDBA, we do not increase the node length globally.

As noted in the previous subsection, repeats correspond to nodes in the sequence graph we call *X-nodes*. Here the converse is false: not all repeats correspond to X-nodes. A repeat is said to be *all-bridged *if *all *repeat copies are bridged, and an X-node is called all-bridged if the corresponding repeat is all-bridged.

**Figure 8:**
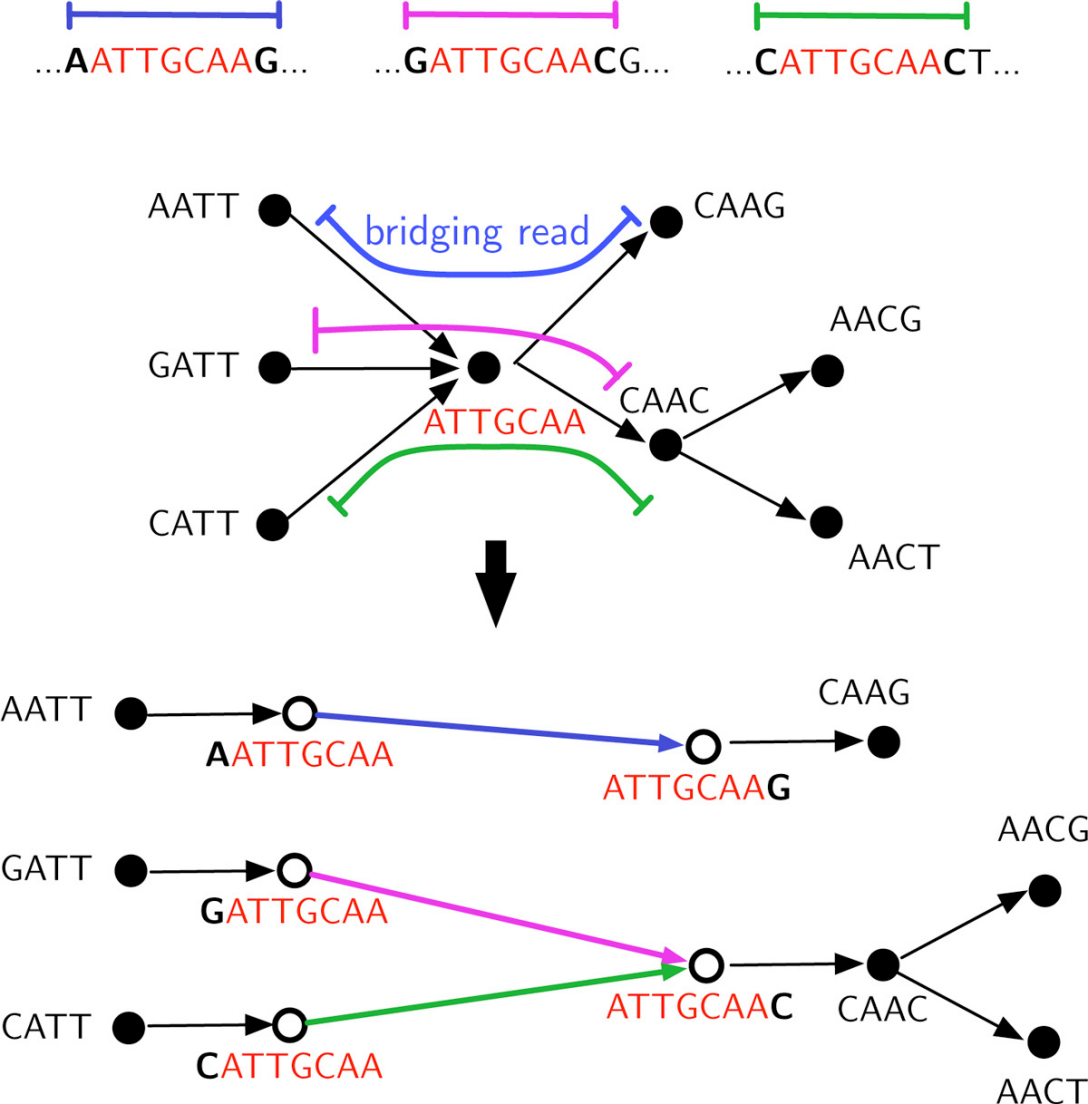
**MULTIBRIDGING resolves an X-node with label ATTGCAA corresponding to a triple repeat**.

The requirement that triple repeats be all bridged allows them to be resolved *locally *(Figure 8). The X-node resolution procedure given in Step 4 of MULTIBRIDGING can be interpreted in the *K*-mer graph framework as increasing *K *locally so that repeats do not appear in the graph. In order to do this, we introduce the following notation for extending nodes: Given an edge (**v**, **q**) with weight *a*_**v**,**q**_, let **v**^→**q**^denote **v **extended one base to the right along (**v**, **q**), i.e. ${\text{v}}^{\to \text{q}}={\text{vq}}_{a_{vq}+1}^{1}$ (notation introduced in Sec.). Similarly, let ${}^{p\to }\text{v}={\text{p}}_{\text{end}-a_{pv}}^{1}\text{v}$. MULTIBRIDGING is described as follows.

**Algorithm 1 **MULTIBRIDGING. Input: reads R , parameter *K*. Output: sequence $\hat{\text{s}}$.

K-mer steps 1-3:

1. For each subsequence **x **of length *K *in a read, form a node with label **x**.

2. For each read, add edges between nodes representing adjacent *K*-mers in the read.

3. Condense the graph (c.f. Figure 6).

4. *Bridging step: *(See Figure 8). While there exists a bridged X-node **v**: (i) For each edge (**p***_i_*, **v**) with weight $a_{p_{i,}v}$, create a new node ${\text{u}}_{i}{=}^{{\text{p}}_{i}\to }v$ and an edge (**p***_i_*, **u***_i_*) with weight $1+a_{p_{i,}v}$. Similarly for each edge (**v**, **q***_j _*), create a new node $w_{j}=v^{\to q_{j}}$ and edge (**w**_j _, **q***_j _*). (ii) If **v **has a self-loop (**v**, **v**) with weight *a*_**v**,**v**_, add an edge $\left(v^{\to v}{,}^{v\to }v\right)$ with weight *a*_**v**,**v **_+ 2. (iii) Remove node **v **and all incident edges. (iv) For each pair **u***_i_*, **w***_j _*adjacent in a read, add edge (**u***_i_*,**w***_j _*). If exactly one each of the **u***_i _*and **w***_j _*nodes have no added edge, add the edge. (v) Condense graph.

5. *Finishing step: *Find an Eulerian cycle in the graph and return the corresponding sequence.

**Theorem 6**. *The algorithm *MULTIBRIDGING*reconstructs the sequence ***s ***if:*

(a) all interleaved repeats are bridged

(b) all triple repeats are **all-bridged**

*(c) the sequence is covered by the reads*.

A similar analysis as for SIMPLEBRIDGING yields the performance curve of MULTIBRIDGING in Figure 2.

### Gap to lower bound

**Figure 9:**
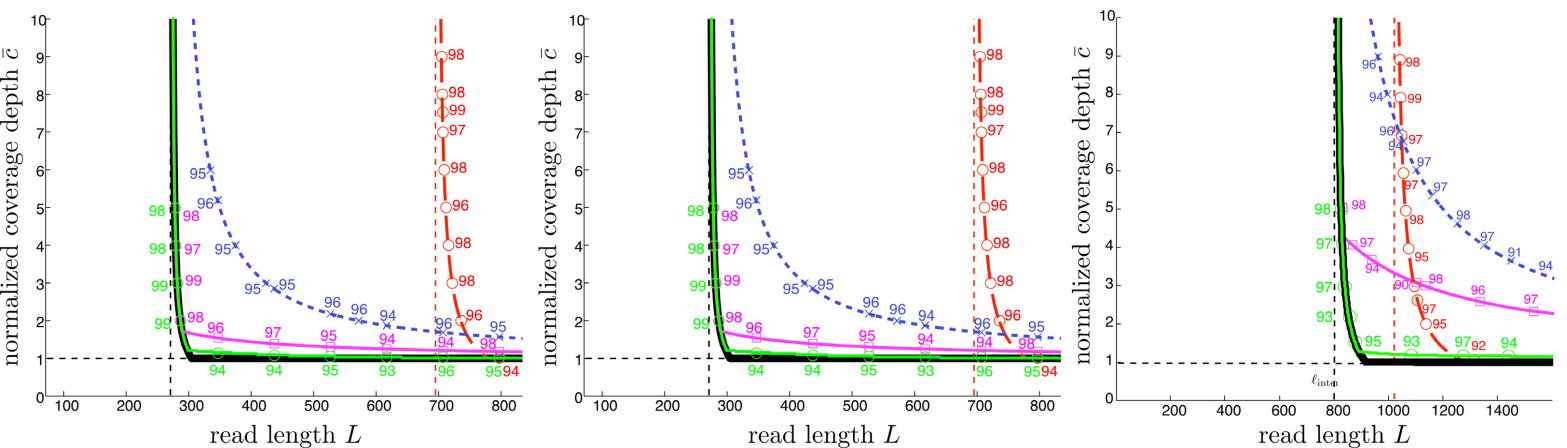
**Simulation results for each of the GAGE reference genomes**. Each simulated (*N,L*) point is marked with the number of correct reconstructions (e.g. 93, 98, 95) on 100 simulated read sets. All four algorithms (GREEDY, DEBRUIJN, SIMPLEBRIDGING, and MULTIBRIDGING) were run on *S. Aureus*, *R. sphaeroides *and hc14. Note that MULTIBRIDGING is very close to the lower bound on all 3 datasets.

The only difference between the sufficient condition guaranteeing the success of MULTIBRIDGING and the necessary condition of the lower bound is the bridging condition of *triple *repeats: while MULTIBRIDGING requires bridging *all three copies *of the triple repeats, the necessary condition requires only bridging *a single copy*. When *ℓ*_triple _is significantly smaller than *ℓ*_interleaved_, the bridging requirement of interleaved repeats dominates over that of triple repeats and MULTIBRIDGING achieves very close to the lower bound. This occurs in hc19 and the majority of the datasets we looked at. (See Figure 9 and the plots in additional file 1.) A critical phenomenon occurs as *L *increases: for *L < L*_crit _reconstruction is impossible, over a small critical window the bridging requirement of interleaved repeats (primarily the longest) dominates, and then for larger *L*, coverage suffices.

On the other hand, when *ℓ*_triple _is comparable or larger than *ℓ*_interleaved_, then MULTIBRIDGING has a gap in the coverage depth to the lower bound (see for example Figure 3). If we further assume that the longest triple repeat is dominant, then this gap can be calculated to be a factor of $\text{3}\cdot \frac{\text{log}3\varepsilon^{-1}}{\text{log}\varepsilon^{-1}}\approx 3.72$ for *∈ *= 10^-2^. This gap occurs only within the critical window where the repeat-bridging constraint is active. Beyond the critical window, the coverage constraint dominates and MULTIBRIDGING is optimal. Further details are provided in the appendices.

## Simulations and complexity

In order to verify performance predictions, we implemented and ran the algorithms on simulated error-free reads from sequenced genomes. For each algorithm, we sampled (*N, L*) points predicted to give *<*5% error, and recorded the number of times correct reconstruction was achieved out of 100 trials. Figure 9 shows results for the three GAGE reference sequences.

We now estimate the run-time of MULTIBRIDGING. The algorithm has two phases: the *K*-mer graph formation step, and the repeat resolution step. The *K*-mer graph formation runtime can be easily bounded by *O*((*L-K*)*NK*), assuming *O*(*K*) look-up time for each of the (*L-K*)*N K*-mers observed in reads. This step is common to all *K*-mer graph based algorithms, so previous works to decrease the practical runtime or memory requirements are applicable.

The repeat resolution step depends on the repeat statistics and choice of *K*. It can be loosely bounded as $O\left(\sum_{\ell =K}^{L}L\sum_{\begin{array}{c} \text{max}\;\;\text{repeats}\;\;x\\ \text{of}\;\;\text{length}\;\;\ell \end{array}}\;\;d_{x}\right)$. The second sum is over distinct of length maximal repeats *x *of length *ℓ* and *d_x _*is the number of (not necessarily maximal) copies of repeat *x*. The bound comes from the fact that each maximal repeat of length *K < ℓ < L *is resolved via exactly one bridged X-node, and each such resolution requires examining at most the *Ld_x _*distinct reads that contain the repeat. We note that $\sum_{\ell =K}^{L}L\sum_{\begin{array}{c} \text{max}\;\;\text{repeats}\;\;x\\ \text{of}\;\;\text{length}\;\;\ell \end{array}}\;\;d_{x}<L\sum_{\ell =K}^{L}a_{\ell }$, and the latter quantity is easily computable from our sufficient statistics.

For our data sets, with appropriate choice of *K*, the bridging step is much simpler than the *K*-mer graph formation step: for *R. sphaeroides *we use *K *= 40 to get $\sum_{\ell =K}^{L}La_{\ell }=412$; in contrast, *N >*22421 for the relevant range of *L*. Similarly, for hc14, using *K *= 300, $\sum_{\ell =K}^{L}La_{\ell }=661$ while *N >*733550; for *S. Aureus*, $\sum_{\ell =K}^{L}La_{\ell }=558$ while *N >*8031.

## Discussions and extensions

The notion of *optimal shotgun assembly *is not commonly discussed in the literature. One reason is that there is no universally agreed-upon metric of success. Another reason is that most of the optimization-based formulations of assembly have been shown to be NP-hard, including Shortest Common Superstring [24], [5], De Bruijn Superwalk [8], [25], and Minimum s-Walk on the string graph [9], [25]. Thus, it would seem that optimal assembly algorithms are out of the question from a computational perspective. What we show in this paper is that if the goal is complete reconstruction, then one can define a clear notion of optimality, and moreover there is a computationally efficient assembly algorithm (MULTIBRIDGING) that is near optimal for a wide range of DNA repeat statistics. So while the reconstruction problem may well be NP-hard, typical instances of the problem seem much easier than the worst-case, a possibility already suggested by Nagarajan and Pop [26].

The MULTIBRIDGING algorithm is near optimal in the sense that, for a wide range of repeat statistics, it requires the minimum read length and minimum coverage depth to achieve complete reconstruction. However, since the repeat statistics of a genome to be sequenced are usually not known in advance, this minimum required read length and minimum required coverage depth may also not be known in advance. In this context, it would be useful for the Multi-Bridging algorithm to *validate *whether its assembly is correct. More generally, an interesting question is to seek algorithms which are not only optimal in their data requirements but also provide a measure of confidence in their assemblies.

How realistic is the goal of complete reconstruction given current-day sequencing technologies? The minimum read lengths *L*_crit _required for complete reconstruction on the datasets we examined are typically on the order of 500-3000 base pairs (bp). This is substantially longer than the reads produced by Illumina, the current dominant sequencing technology, which produces reads of lengths 100-200bp; however, other technologies produce longer reads. PacBio reads can be as long as several thousand base pairs, and as demonstrated by [27], the noise can be cleaned by Illumina reads to enable near complete reconstruction. Thus our framework is already relevant to some of the current cutting edge technologies. To make our framework more relevant to short-read technologies such as Illumina, an important direction is to incorporate mate-pairs in the read model, which can help to resolve long repeats with short reads. Other extensions to the basic shotgun sequencing model: **heterogenous read lengths**: This occurs in some technologies where the read length is random (e.g. Pacbio) or when reads from multiple technologies are used. Generalized Ukkonen's conditions and the sufficient conditions of MULTIBRIDGING extend verbatim to this case, and only the computation of the bridging probability (3) has to be slightly modified.

**non-uniform read coverage**: Again, only the computation of the bridging probability has to be modified. One issue of interest is to investigate whether reads are sampled less frequently from long repeat regions. If so, our framework can quantify the performance hit.

**double strand**: DNA is double-stranded and consists of a length-*G *sequence **u **and its reverse complement $\tilde{u}$. Each read is either sampled from **u **or $\tilde{u}$. This more realistic scenario can be mapped into our single-strand model by defining **s **as the length-2*G *concatenation of **u **and $\tilde{u}$, transforming each read into itself and its reverse complement so that there are 2*N *reads. Generalized Ukkonen's conditions hold verbatim for this problem, and MULTIBRIDGING can be applied, with the slight modification that instead of looking for a single Eulerian path, it should look for two Eulerian paths, one for each component of the sequence graph after repeat-resolution. An interesting aspect of this model is that, in addition to interleaved repeats on the single strand **u**, *reverse complement repeats *on **u **will also induce interleaved repeats on the sequence **s**.

## Author's contributions

Author ordering is alphabetical. GB, MB, and DT developed the method, performed the mathematical analysis, and wrote the manuscript. MB and GB implemented the method. MB designed and carried out the simulations. All authors read and approved the final manuscript.

## Competing interests

The authors declare that they have no competing interests.

## Supplementary Material

Additional file 1 In this supplementary material, we display in Figures 10-17 the output of our pipeline for 9 datasets (in addition to hc19, whose output is in the introduction, and the GAGE datasets R. sphaeroides, S. Aureus, and hc14). For each dataset we plot
$$\text{log}\left(1+a_{\ell }\right),$$
the log of one plus the number of repeats of each length *ℓ*. From the repeat statistics *a_m_*, *b_m,n_*, and *c_m_*, we produce a feasibility plot. The thick black line denotes the lower bound on feasible *N, L*, and the green line is the performance achieved by MULTIBRIDGING. Click here for file

## Declarations

Publication of this article is funded by the NSF Center for Science of Information grant agreement CCF-0939370.
