# Supplementary material for: Optimal assembly for high throughput shotgun sequencing
Source: BMC Bioinformatics. 2013 Jul 9;14(Suppl 5):S18. doi: 10.1186/1471-2105-14-S5-S18 (PMC3706340; doi:10.1186/1471-2105-14-S5-S18)
Supplement: Additional file 1 — In this supplementary material, we display in Figures 10-17 the output of our pipeline for 9 datasets (in addition to hc19, whose output is in the introduction, and the GAGE datasets R. sphaeroides, S. Aureus, and hc14). For each dataset we plot log(1+aℓ), the log of one plus the number of repeats of each length ℓ. From the repeat statistics am, bm,n, and cm, we produce a feasibility plot. The thick black line denotes the lower bound on feasible N, L, and the green line is the performance achieved by MULTIBRIDGING. [file 1471-2105-14-S5-S18-S1.PDF]

Supplementary Material

In this supplementary material, we display in Figures 10–17 the output of our pipeline for 9 datasets (in addition to hc19, whose output is in the introduction, and the GAGE datasets R. sphaeroides, S. Aureus, and hc14). For each dataset we plot

$$\log(1 + a_\ell),$$

the log of one plus the number of repeats of each length ℓ. From the repeat statistics a_m, $b_{m,n}$, and c_m, we produce a feasibility plot. The thick black line denotes the lower bound on feasible N, L, and the green line is the performance achieved by MultiBridging.

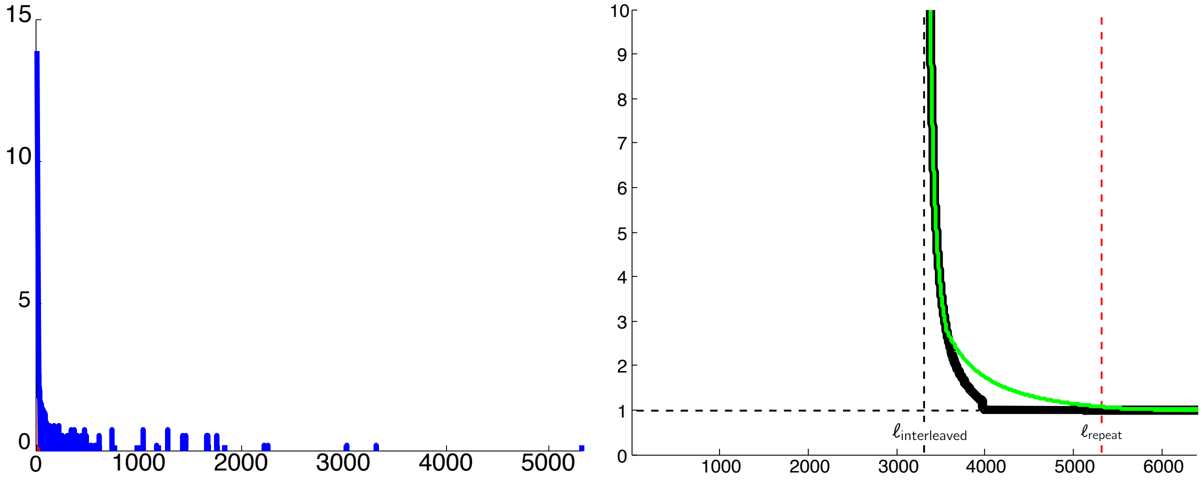

Figure 10: Lactofidus. $G = 2,078,001$, $\ell_{\text{triple}} = 3027$, $\ell_{\text{interleaved}} = 3313$, $\ell_{\text{repeat}} = 5321$.

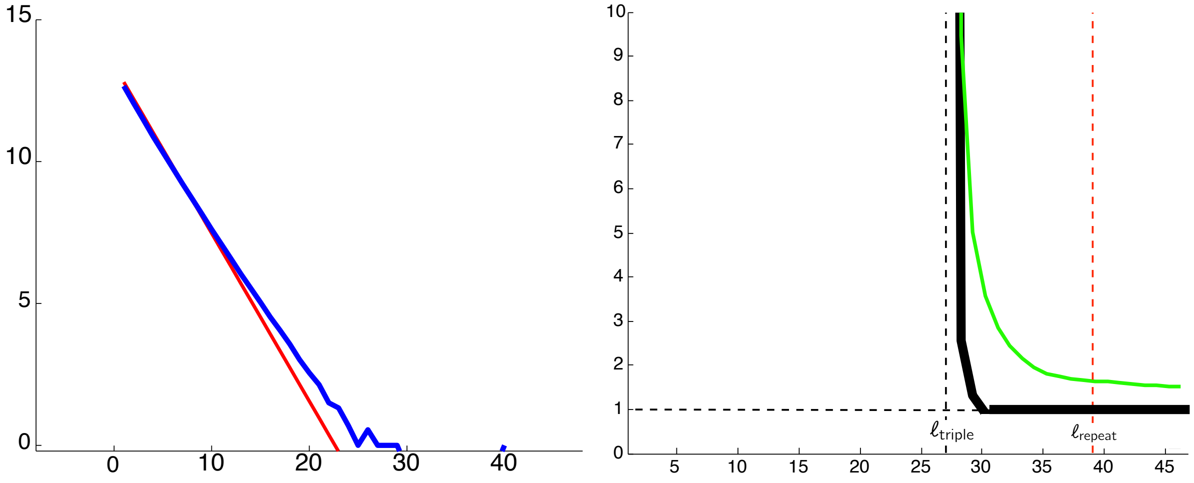

Figure 11: Buchnera. $G = 642,122$, $\ell_{\text{triple}} = 27$, $\ell_{\text{interleaved}} = 23$, $\ell_{\text{repeat}} = 39$.

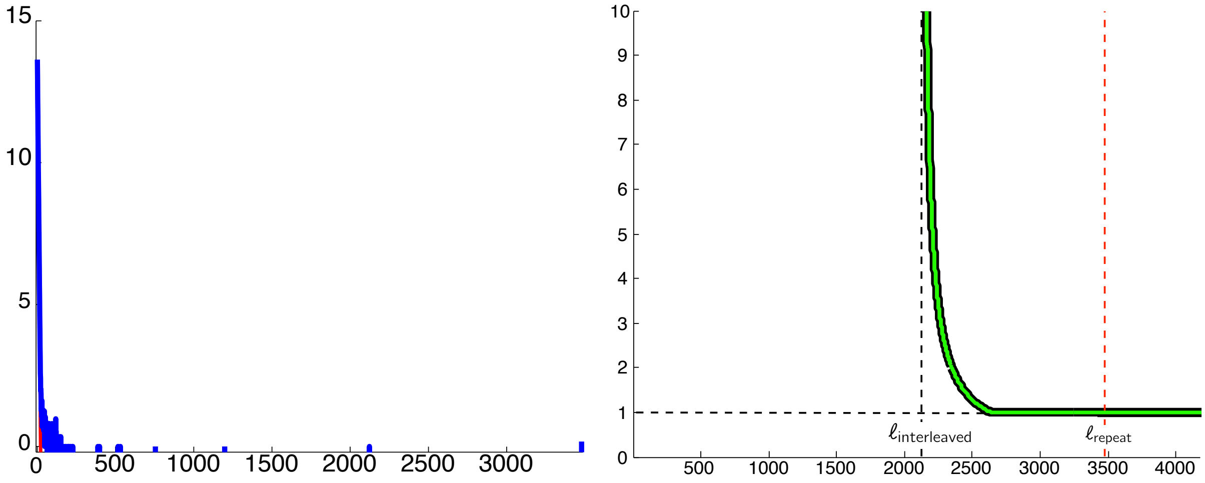

Figure 12: Heli51. $G = 1,589,954$, $\ell_{\text{triple}} = 219$, $\ell_{\text{interleaved}} = 2122$, $\ell_{\text{repeat}} = 3478$.

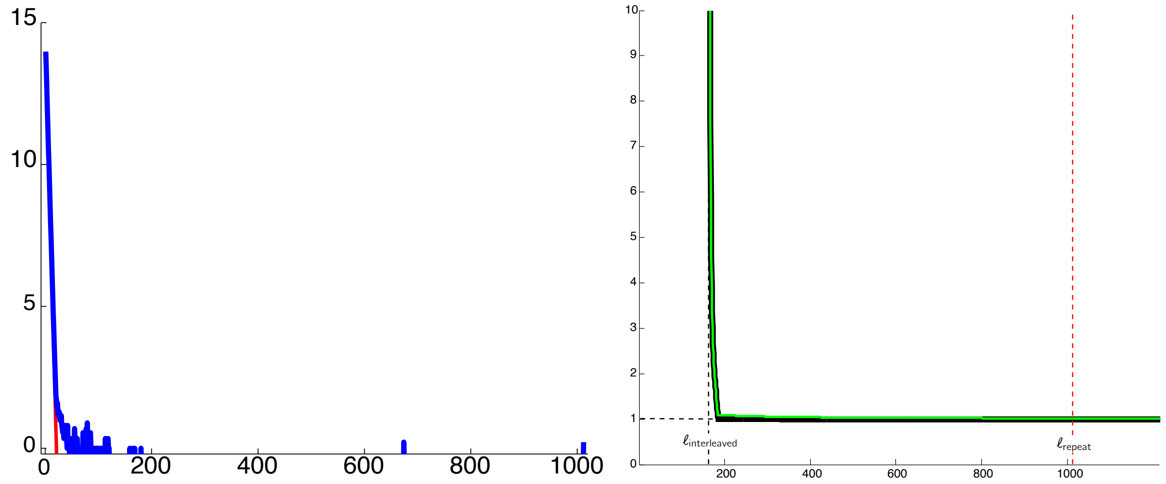

Figure 13: Salmonella. $G = 2,215,568$, $\ell_{\text{triple}} = 112$, $\ell_{\text{interleaved}} = 163$, $\ell_{\text{repeat}} = 1011$.

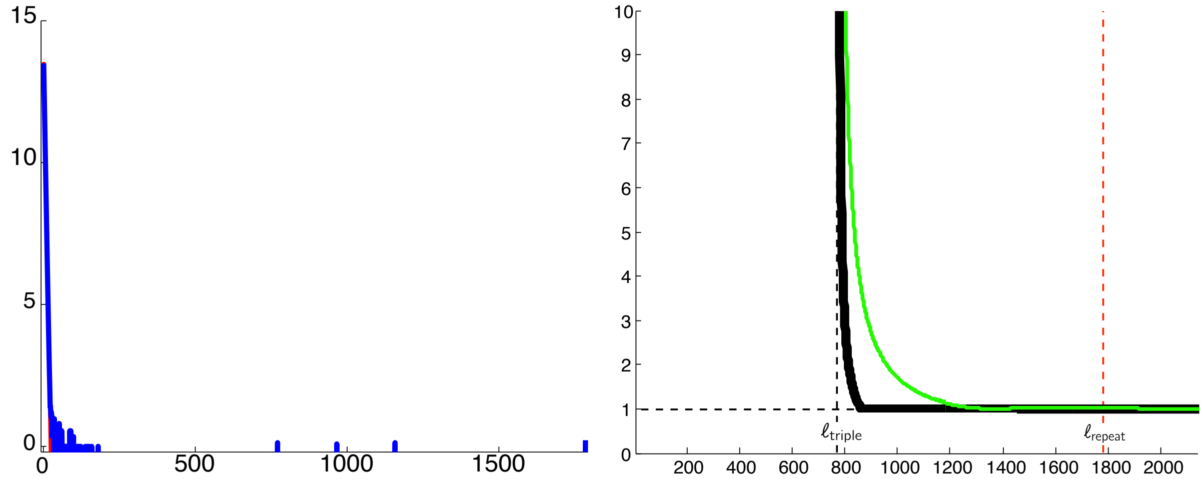

Figure 14: Perkinsus marinus. $G = 1,440,372$, $\ell_{\text{triple}} = 770$, $\ell_{\text{interleaved}} = 92$, $\ell_{\text{repeat}} = 1784$.

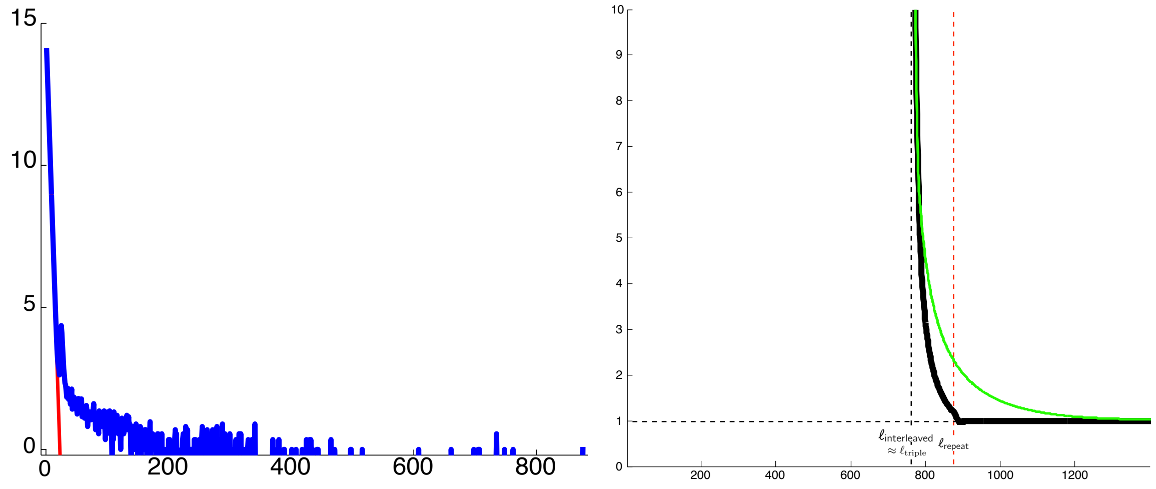

Figure 15: Sulfolobus islandicus. $G = 2,655,198$, $\ell_{\text{triple}} = 734$, $\ell_{\text{interleaved}} = 761$, $\ell_{\text{repeat}} = 875$.

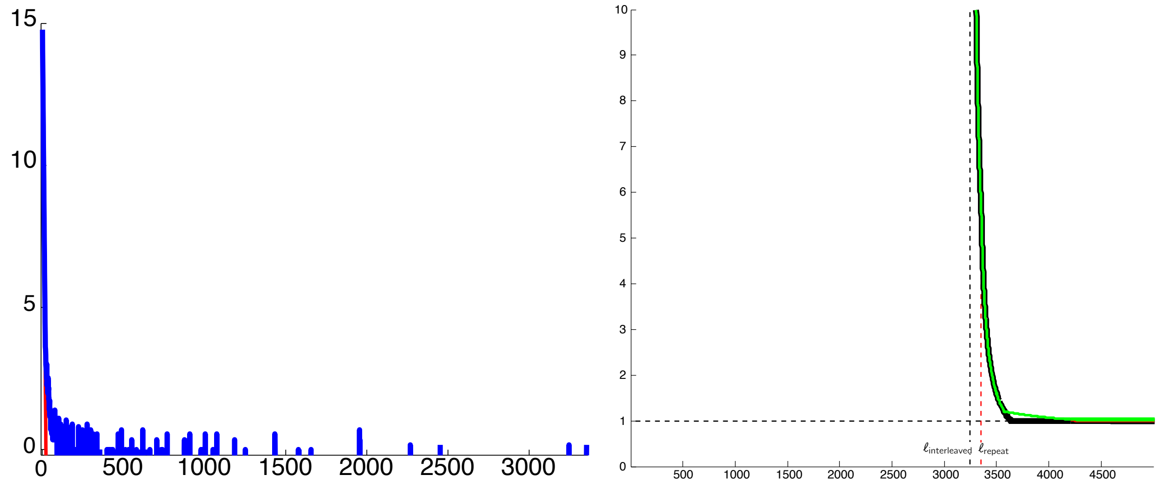

Figure 16: Ecoli536. $G = 4,938,920$, $\ell_{\text{triple}} = 2267$, $\ell_{\text{interleaved}} = 3245$, $\ell_{\text{repeat}} = 3353$.

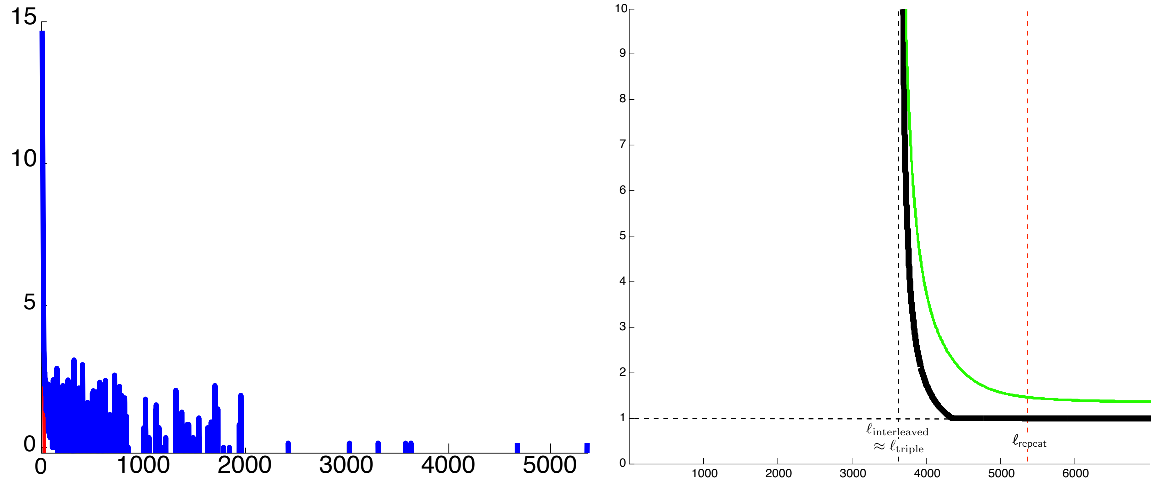

Figure 17: Yesnina. $G = 4,504,254$, $\ell_{\text{triple}} = 3573$, $\ell_{\text{interleaved}} = 3627$, $\ell_{\text{repeat}} = 5358$.

Lower bounds on coverage depth

The lower bounds are based on a generalization of Ukkonen's condition to shotgun sequencing, as described in Theorem 1. The proof of Theorem 1 follows by a straightforward modification to the argument in [12] and is omitted here.

Theorem 1. *Given a DNA sequence* $\mathbf{s}$ *and a set of reads, if there is a pair of interleaved repeats or a triple repeat whose copies are all unbridged, then there is another sequence* $\mathbf{s}'$ *of the same length under which the likelihood of observing the reads is the same.*

Lower bound due to interleaved repeats

In this section we derive a necessary condition on N and L in order that the probability of correct reconstruction be at least $1-\epsilon$.

Recall that a pair of repeats, one at positions t_1, t_3 with $t_1 < t_3$ and the second at positions t_2, t_4 with $t_2 < t_4$, is *interleaved* if $t_1 < t_2 < t_3 < t_4$ or $t_2 < t_1 < t_4 < t_3$. From the DNA we may extract a (symmetric) matrix of interleaved repeat statistics b_{mn}, the number of pairs of interleaved repeats of lengths m and n.

We proceed by fixing both N and L and checking whether or not unbridged interleaved repeats occur with probability higher than ϵ. We will break up repeats into 2 categories: repeats of length at least $L-1$ (these are always unbridged), and repeats of length less than $L-1$ (these are sometimes unbridged). We assume that $L > \ell_{\text{interleaved}} + 1$, or equivalently $b_{ij} = 0$ for all $i, j \geq L-1$, since otherwise there are (with certainty) unbridged interleaved repeats and Ukkonen's condition is violated.

First, we estimate the probability of error due to interleaved repeats of lengths $i < L-1$ and $j \geq L-1$. The repeat of length j is too long to be bridged, so an error occurs if the repeat of length i is unbridged. For a repeat, as long as the two copies' locations are not too nearby[1], each copy is bridged independently and hence the probability that both copies of the repeat of length i are unbridged is $p_i^{\text{unbridged}} = e^{-2\frac{N}{G}(L-i-1)}$. (Recall that a repeat is unbridged if *both* copies are unbridged.)

A union bound estimate[2] gives a probability of error

$$P_{\text{error}} \approx \frac{1}{2} \sum_{\substack{m<L-1 \\ n \geq L-1}} b_{mn} e^{-2\lambda(L-m-1)} . \tag{8}$$

Requiring the error probability to be less than ϵ and solving for L gives the necessary condition

$$L \geq \frac{1}{2\lambda} \log \frac{\gamma_1}{2\epsilon} = \frac{G}{2N} \log \frac{\gamma_1}{2\epsilon} , \tag{9}$$

where $\gamma_1 := \sum_{\substack{m<L-1 \\ n \geq L-1}} b_{mn} e^{2(N/G)(m+1)}$ is a simple function of the interleaved repeat statistic b_{mn}.

We now estimate the probability of error due to interleaved repeat pairs in which both repeats are shorter than $L-1$. In this case only one repeat of each interleaved repeat pair must be bridged. Again a union bound estimate gives

$$P_{\text{error}} \approx \frac{1}{2} \sum_{m,n<L-1} b_{mn} e^{-2\lambda(L-m-1)} e^{-2\lambda(L-n-1)} .$$

[1]More precisely, for the two copies of a a repeat of length ℓ to be bridged independently requires that no single read can bridge them both. This means their locations t and $t+d$ must have separation $d \geq L-\ell-2$.

[2]The union bound on probabilities gives an *upper bound*, so its use here is only an approximation. To get a rigorous lower bound we can use the inclusion-exclusion principle, but the difference in the two computations is negligible for the data we observed. For ease of exposition we opt to present the simpler union bound estimate.

Requiring the error probability to be less than ϵ gives the necessary condition

$$L \geq \frac{1}{4\lambda} \log \frac{\gamma_2}{2\epsilon} = \frac{G}{4N} \log \frac{\gamma_2}{2\epsilon}, \qquad (10)$$

where $\gamma_2 := \sum_{m,n<L-1} b_{mn} e^{2(N/G)(m+n+2)}$ and similarly to γ_1 is computed from b_{mn}.

Lower bound due to triple repeats

We translate the generalized Ukkonen's condition prohibiting unbridged triple repeats into a condition on L and N. Let c_m denote the number of triple repeats of length m. Then a union bound estimate gives

$$\mathbb{P}(\mathcal{E}) \approx \frac{1}{2} \sum_m c_m e^{-3\lambda(L-m-1)}. \qquad (11)$$

Requiring $\mathbb{P}(\mathcal{E}) \leq \epsilon$ and solving for L gives

$$L \geq \frac{1}{3\lambda} \log \frac{\gamma_3}{2\epsilon} = \frac{G}{3N} \log \frac{\gamma_3}{2\epsilon}, \qquad (12)$$

where $\gamma_3 := \sum_m c_m e^{3(N/G)(m+1)}$.

Remark 7. As discussed here and in Section , if the DNA sequence is not covered by the reads or there are unbridged interleaved or triple repeats, then reconstruction is not possible. But there is another situation which must be ruled out. Without knowing its length a priori, it is impossible to know how many copies of the DNA sequence are actually present: if the sequence $\mathbf{s}$ to be assembled consists of multiple concatenated copies of a shorter sequence, rather than just one copy, the probability of observing any set of reads will be the same. Since it is unlikely that a true DNA sequence will consist of the same sequence repeated multiple times, we assume this is not the case throughout the paper. Equivalently, if $\mathbf{s}$ does consist of multiple concatenated copies of a shorter sequence, we are content to reconstruct a single copy. If available, knowledge of the approximate length of $\mathbf{s}$ would then allow to reconstruct.

Proofs for algorithms

Proof of Theorem 2 (Greedy)

The greedy algorithm's underlying data structure is the overlap graph, where each node represents a read and each (directed) edge $(\mathbf{x}, \mathbf{y})$ is labeled with the overlap $\text{ov}(\mathbf{x}, \mathbf{y})$ (defined as the the length of the shared prefix/suffix) between the incident nodes' reads. For a node $\mathbf{v}$, the in-degree [out-degree] is the number of edges in the graph directed towards [away from] $\mathbf{v}$. The greedy algorithm is described as follows.

Algorithm 2 GREEDY. Input: reads $\mathcal{R}$. Output: sequence $\hat{\mathbf{s}}$.

1. For each read with sequence $\mathbf{x}$, form a node with label $\mathbf{x}$.

Greedy steps 2-3:

2. Consider all pairs of nodes $\mathbf{x}_1, \mathbf{x}_2$ in $\mathbb{G}$ satisfying $d_{\text{out}}(\mathbf{x}_1) = d_{\text{in}}(\mathbf{x}_2) = 0$, and add an edge $(\mathbf{x}_1, \mathbf{x}_2)$ with largest value $\text{ov}(\mathbf{x}_1, \mathbf{x}_2)$.
3. Repeat Step 2 until no candidate pair of nodes remains.

Finishing step:

4. Output the sequence corresponding to the unique cycle in $\mathbb{G}$.

Theorem 2. *Given a sequence* $\mathbf{s}$ *and a set of reads,* GREEDY *returns* $\mathbf{s}$ *if every repeat is bridged.*

Proof. We prove the contrapositive. Suppose GREEDY makes its first error in merging reads $\mathbf{r}_i$ and $\mathbf{r}_j$ with overlap $\text{ov}(\mathbf{r}_i, \mathbf{r}_j) = \ell$. Now, if $\mathbf{r}_j$ is the successor to $\mathbf{r}_i$, then the error is due to incorrectly aligning the reads; the other case is that $\mathbf{r}_j$ is not the successor of $\mathbf{r}_i$. In the first case, the subsequence $\mathbf{s}_{t_j}^{\ell}$ is repeated at location $\mathbf{s}_{t_i+L-\ell}^{\ell}$, and no read bridges either repeat copy.

In the second case, there is a repeat $\mathbf{s}_{t_j}^{\ell} = \mathbf{s}_{t_i+L-\ell}^{\ell}$. If $\mathbf{s}_{t_i+L-\ell}^{\ell}$ is bridged by some read $\mathbf{r}_k$, then $\mathbf{r}_i$ has overlap at least $\ell + 1$ with $\mathbf{r}_k$, implying that read $\mathbf{r}_i$ has already found its successor before step ℓ (either $\mathbf{r}_k$ or some other read with even higher overlap). A similar argument shows that $\mathbf{s}_{t_j}^{\ell}$ cannot be bridged, hence there is an unbridged repeat. □

Proofs for K-mer algorithms

Background

We give some mathematical background leading to the proof of Theorem 3 (restated below).

Lemma 8. *Fix an arbitrary K and form the K-mer graph from the $(K+1)$-spectrum $\mathcal{S}_{K+1}$. The sequence $\mathbf{s}$ corresponds to a unique cycle $\mathcal{C}(\mathbf{s})$ traversing each edge at least once.*

To prove the lemma, note that all $(K+1)$-mers in $\mathbf{s}$ correspond to edges and adjacent $(K+1)$-mers in $\mathbf{s}$ are represented by adjacent edges. An induction argument shows that $\mathbf{s}$ corresponds to a cycle. The cycle traverses all the edges, since each edge represents a unique $(K+1)$-mer in $\mathbf{s}$.

In both SBH and shotgun sequencing the number of times each edge e is traversed by $\mathcal{C}(\mathbf{s})$ (henceforth called the *multiplicity* of e) is unknown a priori, and finding this number is part of the reconstruction task. Repeated $(K+1)$-mers in $\mathbf{s}$ correspond to edges in the K-mer graph traversed more than once by $\mathcal{C}(\mathbf{s})$, i.e. having multiplicity greater than one. In order to estimate the multiplicity, previous works seek a solution to the so-called Chinese Postman Problem (CPP), in which the goal is to find a cycle of the shortest total length traversing every edge in the graph (see e.g. [28], [16], [8], [7]). It is not obvious under what conditions the CPP solution *correctly* assigns multiplicities in agreement with $\mathcal{C}(\mathbf{s})$. For our purposes, as we will see in Theorem 3, the multiplicity estimation problem can be sidestepped (thereby avoiding solving CPP) through a modification to the K-mer graph.

Ignoring the issue of edge multiplicities for a moment, Pevzner [18] showed for the SBH model that if the edge multiplicities are known with multiple copies of each edge included according to the multiplicities, and moreover Ukkonen's condition is satisfied, then there is a *unique Eulerian cycle* in the K-mer graph and the Eulerian cycle corresponds to the original sequence. (An Eulerian cycle is a cycle traversing each edge exactly once.) Pevzner's algorithm is thus to find an Eulerian cycle and read off the corresponding sequence. Both steps can be done efficiently.

Lemma 9 (Pevzner [18])**.** *In the SBH setting, if the edge multiplicities are known, then there is a unique Eulerian cycle in the K-mer graph with $K = L - 1$ if and only if there are no unbridged interleaved repeats or unbridged triple repeats.*

Most practical algorithms (e.g. [16], [22], [23]) condense unambiguous paths (called unitigs by Myers [29] in a slightly different setting) for computational efficiency. The more significant benefit for us, as shown in Theorem 3, is that if Ukkonen's condition is satisfied then condensing the graph obviates the need to estimate multiplicities. Condensing a K-mer graph results in a graph of the following type.

Definition 10 (Sequence graph)**.** A *sequence graph* is a graph in which each node is labeled with a subsequence, and edges $(\mathbf{u}, \mathbf{v})$ are labeled with an *overlap* $a_{\mathbf{uv}}$ such the subsequences $\mathbf{u}$ and $\mathbf{v}$ overlap by $a_{\mathbf{uv}}$ (the overlap is not necessarily maximal). In other words, an edge label $a_{\mathbf{uv}}$ on $e = (\mathbf{u}, \mathbf{v})$ indicates that the $a_{\mathbf{uv}}$-length suffix of $\mathbf{u}$ is equal to the $a_{\mathbf{uv}}$-length prefix of $\mathbf{v}$.

The sequence graph generalizes both the overlap graph used by Greedy in Section (nodes correspond to reads, and edge overlaps are *maximal* overlaps) as well as the K-mer algorithms discussed in this section (nodes correspond to K-mers, and edge overlaps are $K - 1$).

In order to speak concisely about concatenated sequences in the sequence graph, we extend the notation $\mathbf{s}_t^\ell$ (denoting the length-ℓ subsequence of the DNA sequence $\mathbf{s}$ starting at position t) which was introduced in Section ; we abuse notation slightly, and write $\mathbf{s}_t^{\text{end}}$ to indicate the subsequence of $\mathbf{s}$ starting at position t and having length so that its end coincides with the end of $\mathbf{s}$.

We will perform two basic operations on the sequence graph. For an edge $e = (\mathbf{u}, \mathbf{v})$ with overlap $a_{\mathbf{uv}}$, *merging* $\mathbf{u}$ and $\mathbf{v}$ along e produces the concatenation $\mathbf{u}_1^{\text{end}}\mathbf{v}_{a_{\mathbf{uv}}+1}^{\text{end}}$. *Contracting* an edge $e = (\mathbf{u}, \mathbf{v})$ entails two steps (c.f. Fig. 6): first, merging $\mathbf{u}$ and $\mathbf{v}$ along e to form a new node $\mathbf{w} = \mathbf{u}_1^{\text{end}}\mathbf{v}_{a_{\mathbf{uv}}+1}^{\text{end}}$, and, second, edges to $\mathbf{u}$ are replaced with edges to $\mathbf{w}$, and edges from $\mathbf{v}$ are replaced by edges from $\mathbf{w}$. We will only contract edges $(\mathbf{u}, \mathbf{v})$ with $d_{\text{out}}(\mathbf{u}) = d_{\text{in}}(\mathbf{v}) = 1$.

The condensed graph is defined next.

Definition 11 (Condensed sequence graph)**.** The *condensed sequence graph* replaces unambiguous paths by single nodes. Concretely, any edge $e = (u, v)$ with $d_{\text{out}}(u) = d_{\text{in}}(v) = 1$ is contracted, and this is repeated until no candidate edges remain.

For a path $\mathcal{P} = \mathbf{v}_1, \mathbf{v}_2, \ldots, \mathbf{v}_q$ in the original graph, the corresponding path in the condensed graph is obtained by contracting an edge $(\mathbf{v}_i, \mathbf{v}_{i+1})$ whenever it is contracted in the graph, replacing the node $\mathbf{v}_1$ by $\mathbf{w}$ whenever an edge $(\mathbf{u}, \mathbf{v}_1)$ is contracted to form $\mathbf{w}$, and similarly for the final node $\mathbf{v}_q$. It is impossible for an intermediate node $\mathbf{v}_i$, $2 \leq i < q$, to be merged with a node outside of $\mathcal{P}$, as this would violate the condition $d_{\text{out}}(u) = d_{\text{in}}(v) = 1$ for edge contraction in Defn. 11.

In the condensed sequence graph $\mathbb{G}$ obtained from a sequence $\mathbf{s}$, nodes correspond to subsequences via their labels, and paths in $\mathbb{G}$ correspond to subsequences in $\mathbf{s}$ via merging the constituent nodes along the path. If the subsequence corresponding to a node $\mathbf{v}$ appears twice or more in $\mathbf{s}$, we say that $\mathbf{v}$ corresponds to a repeat. Conversely, subsequences of length $\ell \geq K$ in $\mathbf{s}$ correspond to paths $\mathcal{P}$ of length $\ell - K + 1$ in the K-mer graph, and thus by the previous paragraph also to paths in the condensed graph $\mathbb{G}$.

We record a few simple facts about the condensed sequence graph obtained from a K-mer graph.

Lemma 12. *Let $\mathbb{G}_0$ be the K-mer graph constructed from the $(K+1)$-spectrum of $\mathbf{s}$ and let $\mathcal{C}_0 = \mathcal{C}_0(\mathbf{s})$ be the cycle corresponding to $\mathbf{s}$. In the condensed graph $\mathbb{G}$, let $\mathcal{C}$ be the cycle obtained from $\mathcal{C}_0$ by contracting the same edges as those contracted in $\mathbb{G}_0$.*

1. *Edges in $\mathbb{G}_0$ can be contracted in any order, resulting in the same graph $\mathbb{G}$, so the condensed graph is well-defined. Similarly $\mathcal{C}$ is well-defined.*
2. *The cycle $\mathcal{C}$ in $\mathbb{G}$ corresponds to $\mathbf{s}$ and is the unique such cycle.*
3. *The cycle $\mathcal{C}$ in $\mathbb{G}$ traverses each edge at least once.*

Theorem 3. *Let $\mathcal{S}_{K+1}$ be the $(K+1)$-spectrum of $\mathbf{s}$ and $\mathbb{G}_0$ be the K-mer graph constructed from $\mathcal{S}_{K+1}$, and let $\mathbb{G}$ be the condensed sequence graph obtained from $\mathbb{G}_0$. If Ukkonen's condition is satisfied, i.e. there are no triple repeats or interleaved repeats of length at least K, then there is a unique Eulerian cycle $\mathcal{C}$ in $\mathbb{G}$ and $\mathcal{C}$ corresponds to $\mathbf{s}$.*

Proof. We will show that if Ukkonen's condition is satisfied, the cycle $\mathcal{C} = \mathcal{C}(\mathbf{s})$ in $\mathbb{G}$ corresponding to $\mathbf{s}$ (constructed in Lemma 12) traverses each edge exactly once in the condensed K-mer graph, i.e. $\mathcal{C}$ is Eulerian. Pevzner's [18] arguments show that if there are multiple Eulerian cycles then Ukkonen's condition is violated, so it is sufficient to prove that $\mathcal{C}$ is Eulerian. As noted in Lemma 12, $\mathcal{C}$ traverses each edge at least once, and thus it remains only to show that $\mathcal{C}$ traverses each edge *at most* once.

To begin, let $\mathcal{C}_0$ be the cycle corresponding to $\mathbf{s}$ in the original K-mer graph $\mathbb{G}_0$. We argue that every edge $(\mathbf{u}, \mathbf{v})$ traversed twice by $\mathcal{C}_0$ in the K-mer graph $\mathbb{G}_0$ has been contracted in the condensed graph $\mathbb{G}$ and hence in $\mathcal{C}$. Note that the cycle $\mathcal{C}_0$ does not traverse any node three times in $\mathbb{G}_0$, for this would imply the existence of a triple repeat of length K, violating the hypothesis of the Lemma. It follows that the node $\mathbf{u}$ cannot have two outgoing edges in $\mathbb{G}_0$ as $\mathbf{u}$ would then be traversed three times; similarly, $\mathbf{v}$ cannot have two incoming edges. Thus $d_{\text{out}}(\mathbf{u}) = d_{\text{in}}(\mathbf{v}) = 1$ and, as prescribed in Defn. 11, the edge $(\mathbf{u}, \mathbf{v})$ has been contracted. □

Proofs for SimpleBridging

Since bridging reads extend one base to either end of a repeat, it will be convenient to use the following notation for extending sequences: Given an X-node $\mathbf{v}$ with an incoming edge $(\mathbf{p}, \mathbf{v})$ and an outgoing edge $(\mathbf{v}, \mathbf{q})$, let

$$\mathbf{v}^{\to\mathbf{q}} = \mathbf{v}\,\mathbf{q}^{1}_{a_{\mathbf{vq}}+1}, \quad \text{and} \quad {}^{\mathbf{p}\to}\mathbf{v} = \mathbf{p}^{1}_{\text{end}-a_{\mathbf{pv}}}\mathbf{v}\,. \tag{13}$$

Here $\mathbf{v}^{\to\mathbf{q}}$ denotes the subsequence $\mathbf{v}$ appended with the single next base in the merging of $\mathbf{v}$ and $\mathbf{q}$ and ${}^{\mathbf{p}\to}\mathbf{v}$ the subsequence $\mathbf{v}$ prepended with the single previous base in the merging of $\mathbf{p}$

and $\mathbf{v}$. For example, if $\mathbf{v}$ = ATTC, $\mathbf{p}$ = TCAT, $a_{\mathbf{pv}} = 2$, $\mathbf{q}$ = TTCGCC, and $a_{\mathbf{vq}} = 3$, then $\mathbf{v}^{\to\mathbf{q}}$ = ATTCG, ${}^{\mathbf{p}\to}\mathbf{v}$ = CATTC, and ${}^{\mathbf{p}\to}\mathbf{v}^{\to\mathbf{q}}$ = CATTCG.

The idea is that a bridging read is consistent with only one pair ${}^{\mathbf{p}\to}\mathbf{v}$ and $\mathbf{v}^{\to\mathbf{q}}$ and thus allows to match up edge $(\mathbf{p}, \mathbf{v})$ with $(\mathbf{v}, \mathbf{q})$. This is recorded in the following lemma.

Lemma 13. *Suppose $\mathcal{C}$ corresponds to a sequence $\mathbf{s}$ in a condensed sequence graph $\mathbb{G}$. If a read $\mathbf{r}$ bridges an X-node $\mathbf{v}$, then there are unique edges $(\mathbf{p}, \mathbf{v})$ and $(\mathbf{v}, \mathbf{q})$ such that ${}^{\mathbf{p}\to}\mathbf{v}$ and $\mathbf{v}^{\to\mathbf{q}}$ are adjacent in $\mathbf{r}$.*

SimpleBridging is described as follows.

Algorithm 3 SimpleBridging. Input: reads $\mathcal{R}$, parameter K. Output: sequence $\hat{\mathbf{s}}$.

K-mer steps 1-3:
1. For each subsequence $\mathbf{x}$ of length K in a read, form a node with label $\mathbf{x}$.
2. For each read, add edges between nodes representing adjacent K-mers in the read.
3. Condense the graph as described in Defn. 11.
4. *Bridging step:* See Fig. 7. While there exists an X-node $\mathbf{v}$ with $d_{\text{in}}(\mathbf{v}) = d_{\text{out}}(\mathbf{v}) = 2$ bridged by some read $\mathbf{r}$: (i) Remove $\mathbf{v}$ and edges incident to it. Add duplicate nodes $\mathbf{v}_1, \mathbf{v}_2$. (ii) Choose the unique $\mathbf{p}_i$ and $\mathbf{q}_j$ s.t. ${}^{\mathbf{p}_i\to}\mathbf{v}$ and $\mathbf{v}^{\to\mathbf{q}_j}$ are adjacent in $\mathbf{r}$ and add edges $(\mathbf{p}_i, \mathbf{v}_1)$ and $(\mathbf{v}_1, \mathbf{q}_j)$. Choose the unused $\mathbf{p}_i$ and $\mathbf{q}_j$, add edges $(\mathbf{p}_i, \mathbf{v}_2)$ and $(\mathbf{v}_2, \mathbf{q}_j)$. (iii) Condense the graph.
5. *Finishing step:* Find an Eulerian cycle in the graph and return the corresponding sequence.

Proofs for MultiBridging

In this subsection we recall Theorem 6 stating sufficient conditions for correct reconstruction, and derive the corresponding required coverage depth and read length to meet a target probability of correct reconstruction. The subsection concludes with a proof that the sufficient conditions are correct.

Theorem 6. *The algorithm* MultiBridging *reconstructs the sequence* $\mathbf{s}$ *if:*
(a) all interleaved repeats are bridged

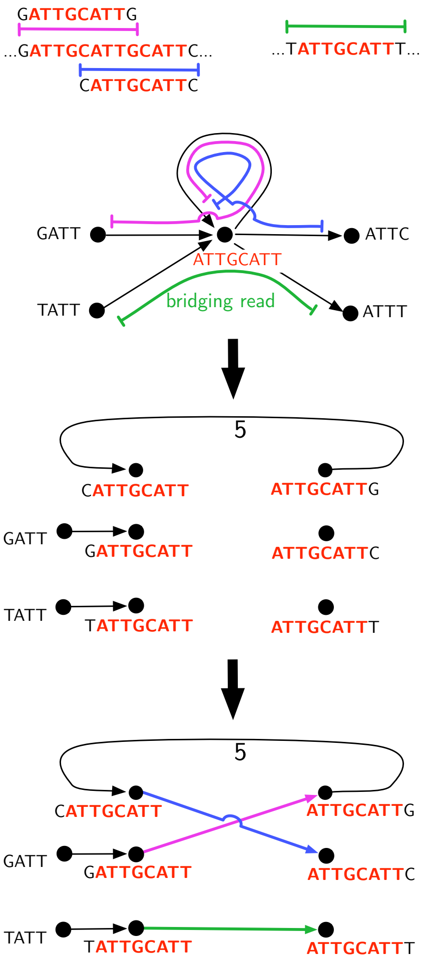

Figure 18: Resolution of X-node with a self-loop.

*(b) all triple repeats are **all-bridged***
(c) the sequence is covered by the reads.

Remark 14. Unlike the previous K-mer algorithms, DeBruijn and SimpleBridging, it is unnecessary to specify a parameter K for MultiBridging. Implicitly MultiBridging uses $K = 1$, which makes the condition that reads overlap by K equivalent to coverage of the genome.

Figure 2 plots the performance of MultiBridging, obtained by solving for the relationship between G, N, L, and ϵ in order to satisfy the conditions of Lemma 6. We first perform the requisite calculations, and then prove the Lemma.

Condition (a) is already dealt with in (9) and (10), and Condition (c) amounts to the requirement that $\frac{N}{N_{\text{LW}}} \geq 1$.

We turn to Condition (b) that all triple repeats are all-bridged. Let c_m denote the number of triple repeats of length m. A union bound estimate over triple repeats for the event that one such triple repeat fails to be all-bridged gives

$$P_{\text{error}} \approx \sum_m 3 \cdot c_m e^{-\lambda(L-m-1)^+}, \qquad (14)$$

and requiring $P_{\text{error}} \leq \epsilon$ and solving for L yields

$$L \geq \frac{1}{\lambda} \log \frac{\gamma_3}{\epsilon} = \frac{G}{N} \log \frac{\gamma_3}{\epsilon}, \qquad (15)$$

where $\gamma_3 := \sum_m 3c_m e^{(N/G)\cdot(m+1)}$ is computed from the triple repeat statistics c_m.

In order to understand the cost of all-bridging triple repeats, compared to simply bridging one copy as required by our lower bound, it is instructive to study the effect of the single longest triple repeat. Setting $c_{\ell_{\text{triple}}} = 1$ and $c_m = 0$ for $m \neq \ell_{\text{triple}}$ makes $\gamma_3 = 3e^{(N/G)\cdot(\ell_{\text{triple}}+1)}$ in (15) and

$$L \geq L_3^{\text{all}} := \ell_{\text{triple}} + 1 + \frac{G}{N} \log 3\epsilon^{-1}. \qquad (16)$$

Bridging the longest triple repeat, as shown in Section , requires

$$L \geq L_3 := \ell_{\text{triple}} + 1 + \frac{G}{3N} \log \epsilon^{-1}. \qquad (17)$$

Solving for N in equations (17) and (16) gives

$$N_3 \geq \frac{G}{3} \cdot \frac{\log \epsilon^{-1}}{L - \ell_{\text{triple}} - 1} \qquad (18)$$

$$N_3^{\text{all}} \geq G \cdot \frac{\log \epsilon^{-1} + \log 3}{L - \ell_{\text{triple}} - 1}. \qquad (19)$$

The ratio is

$$\frac{N_3^{\text{all}}}{N_3} = 3 \cdot \frac{\log 3\epsilon^{-1}}{\log \epsilon^{-1}} \approx 3.72 \quad \text{for } \epsilon = 10^{-2}. \quad (20)$$

This means that if the longest triple repeat is dominant, then for L slightly larger than ℓ_{triple}, MultiBridging needs a coverage depth approximately 3.72 times higher than required by our lower bound.

The remainder of this subsection is devoted to the proving Lemma 6.

We will use $m_{\mathcal{C}}(\mathbf{v})$ to denote the multiplicity (traversal count) a cycle $\mathcal{C}$ assigns a node $\mathbf{v}$. The multiplicity $m_{\mathcal{C}}(\mathbf{v})$ is also equal to the number of times the subsequence $\mathbf{v}$ appears in the sequence corresponding to $\mathcal{C}$. For an edge e, we can similarly let $m_{\mathcal{C}}(e)$ be the number of times $\mathcal{C}$ traverses the edge. The following key lemma relates node multiplicities with the existence of X-nodes.

Lemma 15. *Let $\mathcal{C}$ be a cycle in a condensed sequence graph $\mathbb{G}$, where $\mathbb{G}$ itself is not a cycle, traversing every edge at least once. If $\mathbf{v}$ is a node with maximum multiplicity at least 2, i.e. $m_{\mathcal{C}}(\mathbf{v}) = \max_{u\in\mathbb{G}} m_{\mathcal{C}}(\mathbf{u}) \geq 2$, then $\mathbf{v}$ is an X-node. As a consequence, if $m_{\mathcal{C}}(\mathbf{v}) \geq 3$ for some $\mathbf{v}$, i.e. $\mathcal{C}$ traverses some node at least three times, then $m_{\mathcal{C}}(\mathbf{u}) \geq 3$ for some X-node $\mathbf{u}$.*

Proof. Let $\mathbf{v}$ be a node with maximum multiplicity $m_{\mathcal{C}}(\mathbf{v}) = \max_{\mathbf{u}\in\mathbb{G}} m_{\mathcal{C}}(\mathbf{u})$. We will show that $\mathbf{v}$ is an X-node, i.e. $d_{\text{out}}(\mathbf{v}) \geq 2$ and $d_{\text{in}}(\mathbf{v}) \geq 2$.

We prove that $d_{\text{out}}(\mathbf{v}) \geq 2$ by supposing that $d_{\text{out}}(\mathbf{v}) = 1$ and deriving a contradiction. Denote the outgoing edge from $\mathbf{v}$ by $e = (\mathbf{v}, \mathbf{u})$, where $\mathbf{u}$ is distinct from $\mathbf{v}$ since otherwise $\mathbb{G}$ is a cycle. If $d_{\text{in}}(\mathbf{u}) \geq 2$, then $\mathbf{u}$ must be traversed more times than $\mathbf{v}$, contradicting the maximality of $m_{\mathcal{C}}(\mathbf{v})$, and if $d_{\text{in}}(\mathbf{u}) = 1$, then the existence of the edge e contradicts the fact that $\mathbb{G}$ is condensed. The argument showing that $d_{\text{in}}(\mathbf{v}) \geq 2$ is symmetric to the case $d_{\text{in}}(\mathbf{v}) \geq 2$. □

Proof of Lemma 6. We assume that all triple repeats are all-bridged, that there are no unbridged interleaved repeats, and that all reads overlap their successors by at least 1 base pair. We wish to show that MultiBridging returns the original sequence.

Consider the condensed sequence graph $\mathbb{G}_0$ constructed in steps 1-3 of MultiBridging. Suppose all X-nodes that are either all-bridged or correspond to bridged 2-repeats have been resolved according to repeated application of the procedure in step 4 of MultiBridging, resulting in a condensed sequence graph $\mathbb{G}$. We claim that 1) $\mathbf{s}$ corresponds to a cycle $\mathcal{C}$ in $\mathbb{G}$ traversing every edge at least once, 2) $\mathcal{C}$ is Eulerian, and 3) $\mathcal{C}$ is the unique Eulerian cycle in $\mathbb{G}$.

Proof of Claim 1. Let $\mathbb{G}_n$ be the graph after n resolution steps, and suppose that $\mathcal{C}_n$ is a cycle in $\mathbb{G}_n$ corresponding to the sequence $\mathbf{s}$ and traversing all edges. We will show that there exists a cycle $\mathcal{C}_{n+1}$ in $\mathbb{G}_{n+1}$ corresponding to $\mathbf{s}$ and traversing all edges, and that $\mathbb{G}_t = \mathbb{G}$ for a finite t, so by induction, there exists a cycle $\mathcal{C}$ in $\mathbb{G}$ corresponding to $\mathbf{s}$ and traversing all edges. The base case $n = 0$ was shown in Lemma 8. Moving on to arbitrary $n > 0$, let $\mathbf{v}$ be an X-node in $\mathbb{G}_n$ labeled as in Fig. 6. The X-node resolution step is constructed precisely to preserve the existence of a cycle corresponding to $\mathbf{s}$. Each traversal of $\mathbf{v}$ by the cycle $\mathcal{C}_n$ assigns an incoming edge $(\mathbf{p}_i\mathbf{v})$ to an outgoing edge $(\mathbf{v}, \mathbf{q}_j)$, and the resolution step correctly determines this pairing by the assumption on bridging reads.

Note that all X-nodes in the graph $\mathbb{G}_{n+1}$ continue to correspond to repeats in $\mathbf{s}$. The process terminates: let $\mathcal{L}(\mathbb{G}_i) = \sum_{\mathbf{v}\in\mathbb{G}_i} m_{\mathcal{C}_i}(\mathbf{v})\mathbf{1}_{m_{\mathcal{C}_i}(\mathbf{v})>1}$ and observe that $\mathcal{L}(\mathbb{G}_i)$ is strictly decreasing in i. Thus $\mathbf{s}$ corresponds to a cycle $\mathcal{C}$ in $\mathbb{G}$ traversing each edge at least once.

Proof of Claim 2. We next show that $\mathcal{C}$ is an Eulerian cycle. If $\mathbb{G}$ is itself a cycle, and $\mathbf{s}$ is not formed by concatenating multiple copies of a shorter subsequence (assumed not to be the case, see discussion at end of Section), then $\mathcal{C}$ traverses $\mathbb{G}$ exactly once and is an Eulerian cycle. Otherwise, if $\mathbb{G}$ is not a cycle, then we may apply Lemma 15 to see that any node with $m_{\mathcal{C}}(\mathbf{v}) \geq 3$ implies the existence of an X-node $\mathbf{u}$ with $m_{\mathcal{C}}(\mathbf{u}) \geq 3$. Node $\mathbf{u}$ must be all-bridged, by hypothesis, which means that an additional X-node resolution step can be applied to $\mathbb{G}$, a contradiction. Thus each node $\mathbf{v}$ in $\mathbb{G}$ has multiplicity $m_{\mathcal{C}}(\mathbf{v}) \leq 2$.

We can now argue that no edge $e = (\mathbf{u}, \mathbf{v})$ is traversed twice by $\mathcal{C}$ in the condensed sequence graph $\mathbb{G}$, as it would have been contracted. Suppose $m_{\mathcal{C}}(e) \geq 2$. The node $\mathbf{u}$ cannot have two outgoing edges as this implies $m_{\mathcal{C}}(\mathbf{u}) \geq 3$; similarly, $\mathbf{v}$ cannot have two incoming edges. Thus $d_{\text{out}}(\mathbf{u}) = d_{\text{in}}(\mathbf{v}) = 1$, but by Defn. 11 the edge $e = (\mathbf{u}, \mathbf{v})$ would have been contracted.

Proof of Claim 3. It remains to show that there is a *unique* Eulerian cycle in $\mathbb{G}$. All X-nodes in $\mathbb{G}$ must be unbridged 2-X-nodes (correspond to 2-repeats in $\mathbf{s}$), as all other X-nodes were assumed to be bridged and have thus been resolved in $\mathbb{G}$.

We will map the sequence $\mathbf{s}$ to another sequence $\mathbf{s}'$, allowing us to use the characterization of Lemma 9 for SBH with known multiplicities. Denote by $\mathbb{G}'$ the graph obtained by relabeling each node in $\mathbb{G}$ by a single unique symbol (no matter the original node label length), and setting all edge overlaps to 0. Through the relabeling, $\mathcal{C}$ corresponds to a cycle $\mathcal{C}'$ in $\mathbb{G}'$, and let $\mathbf{s}'$ be the sequence corresponding to $\mathcal{C}'$. Writing $\mathcal{S}_2'$ for the 2-spectrum of $\mathbf{s}'$, the graph $\mathbb{G}'$ is by construction precisely the 1-mer graph created from $\mathcal{S}_2'$, and there is a one-to-one correspondence between X-nodes in $\mathbb{G}'$ and unbridged repeats in $\mathbf{s}'$. Through the described mapping, every unbridged repeat in $\mathbf{s}'$ maps to an unbridged repeat in $\mathbf{s}$, with the *order of repeats preserved*.

There are multiple Eulerian cycles in $\mathbb{G}$ only if there are multiple Eulerian cycles in $\mathbb{G}'$ since the graphs have the same topology, and by Lemma 9 the latter occurs only if there are unbridged interleaved repeats in $\mathbf{s}'$, which by the correspondence in the previous paragraph implies the existence of unbridged interleaved repeats in $\mathbf{s}$. □

Truncation estimate for bridging repeats (Greedy and MultiBridging)

The repeat statistics a_m and c_m used in the algorithm performance curves are potentially overestimates. This is because a large repeat family—one with a large number of copies f—will result in a contribution $\binom{f}{2} \approx f^2/2$ to a_m and $\binom{f}{3} \approx f^3/6$ to c_m.

We focus here on deriving an estimate for the required N, L for bridging all repeats with probability $1-\epsilon$. This upper bound reduces the sensitivity to large families of short repeats. The analogous derivation for all-bridging all triple-repeats is very similar and is omitted.

Suppose there are a_m repeats of length m. The probability that some repeat is unbridged is ap-

proximately, by the union bound estimate,

$$\mathbb{P}(\mathcal{E}) \approx \sum_m a_m e^{-2\lambda(L-m)}\,. \tag{21}$$

Requiring $\mathbb{P}(\mathcal{E}) \le \epsilon$ and solving for L gives

$$L \ge \frac{1}{2\lambda}\log\frac{\gamma}{\epsilon} = \frac{G}{2N}\log\frac{\gamma}{\epsilon}\,, \tag{22}$$

where $\gamma := \sum_m a_m e^{2(N/G)m}$. Now, if a_m *over-counts* the number of repeats for small values of m, the bound in (22) might be loose. In order for each read to overlap the subsequent read by x nucleotides, with probability of failure $\epsilon/2$, it suffices to take

$$L \ge L_{\text{K-cov}}\Big(x, \frac{\epsilon}{2}\Big) := x + \frac{1}{\lambda}\log\frac{2N}{\epsilon}\,. \tag{23}$$

Thus, for any $x < L$, we may replace (22) by

$$L \ge \min_x \max\{\frac{1}{2\lambda}\log\frac{2\gamma(x)}{\epsilon}, L_{\text{K-cov}}(x, \frac{\epsilon}{2})\}\,, \tag{24}$$

where $\gamma(x) = \sum_{m>x} a_m e^{2(N/G)m}$, and obtain a *looser* bound.

Critical window calculations

Window size if $\ell_{\text{interleaved}} \gg \ell_{\text{triple}}$

We focus here on the bound due to interleaved repeats (rather than triple repeats, treated subsequently), and furthermore assume that the effect of the single largest interleaved repeat is dominant. In this case $\ell_{\text{interleaved}} = L_{\text{crit}} - 1$ is the length of the shorter of the pair of interleaved repeats, and let ℓ_1 be the length of the longer of the two. For $L_{\text{crit}} < L \le \ell_1 + 1$, we are in the setting of (9) but with a redefined $\gamma_1 = e^{2(N/G)(L_{\text{crit}}-1)}$. Thus,

$$L \ge L_{\text{crit}} + \frac{G}{2N}\log\epsilon^{-1}\,, \tag{25}$$

and solving for N gives

$$N_{\text{repeat}} = \frac{G}{2}\frac{\log\epsilon^{-1}}{L - \ell_2 - 1} \tag{26}$$

Let L^* be the value of L at which the curve described by constraint (26) intersects the Lander-Waterman coverage value, i.e. $N_{\text{repeat}}(L^*) = N_{\text{LW}}(L^*) := N^*$. This is the minimum read length for which coverage of the sequence suffices for reconstruction.

We now solve for $\frac{L^*}{L_{\text{crit}}}$. First, the Lander-Waterman equation (2) at $N = N^*$ is

$$N^* = \frac{G}{L^*}\log\frac{N^*}{\epsilon}\,, \tag{27}$$

and setting equal the right-hand sides of (27) and (26) at $L = L^*$ gives

$$\frac{G}{L^*}\log\frac{N^*}{\epsilon} = \frac{G}{2}\frac{\log\epsilon^{-1}}{L^* - \ell_2 - 1}\,.$$

A bit of algebra yields

$$\frac{L^*}{L_{\text{crit}}} = \frac{2}{2-x}\,, \tag{28}$$

where

$$x := \cdot\frac{\log\epsilon^{-1}}{\log N^* + \log\epsilon^{-1}}\,. \tag{29}$$

Since $x \le \frac{1}{2}$, equation (28) implies $L^* \le 2L_{\text{crit}}$, and combined with the obvious inequality $L^* \ge L_{\text{crit}}$, we have $L_{\text{crit}} \le L^* \le 2L_{\text{crit}}$. Thus

$$N_{\text{LW}}(2L_{\text{crit}}) \le N^* \le N_{\text{LW}}(L_{\text{crit}})\,, \tag{30}$$

and applying the Lander-Waterman fixed-point equation (2) yet again gives

$$\frac{G}{2L_{\text{crit}}}\log\frac{N_{\text{LW}}(2L_{\text{crit}})}{\epsilon} \le N^* \le \frac{G}{L_{\text{crit}}}\log\frac{N_{\text{LW}}(L_{\text{crit}})}{\epsilon}\,. \tag{31}$$

Writing this out gives

$$\frac{\log\epsilon^{-1}}{\log\frac{G}{L_{\text{crit}}} + \log\log\frac{N_{\text{LW}}(L_{\text{crit}})}{\epsilon} + \log\epsilon^{-1}} \le x \le \frac{\log\epsilon^{-1}}{\log\frac{G}{L_{\text{crit}}} - 1 + \log\log\frac{N_{\text{LW}}(2L_{\text{crit}})}{\epsilon} + \log\epsilon^{-1}}\,,$$

and this can be relaxed to

$$\frac{\log\epsilon^{-1}}{\log\frac{G}{L_{\text{crit}}} + \log\epsilon^{-1} + \log\log\frac{G}{\epsilon L_{\text{crit}}}} \le x \le \frac{\log\epsilon^{-1}}{\log\frac{G}{L_{\text{crit}}} - 1 + \log\epsilon^{-1}}\,. \tag{32}$$

Letting

$$r := \frac{\log\frac{G}{L_{\text{crit}}}}{\log\epsilon^{-1}}\,, \tag{33}$$

we have to a very good approximation

$$\frac{L^*}{L_{\text{crit}}} \approx \frac{2(r+1)}{2(r+1)-1}\,. \tag{34}$$

For $G \sim 10^8$, $L_{\text{crit}} \sim 1000$, and $\epsilon = 5\%$, we get $\log \frac{G}{L_{\text{crit}}} \approx 13.8$ and $\log \epsilon^{-1} \approx 3.0$, so $r \approx 4.6$ and

$$\frac{L^*}{L_{\text{crit}}} = \frac{2(r+1)}{2(r+1)-1} \approx 1.1\,.$$

From (33) we see that the relative size of $\log \epsilon^{-1}$ and $\log \frac{G}{L_{\text{crit}}}$ determines the size of the critical window. If in the previous example $\epsilon = 10^{-5}$, say, then $\frac{L^*}{L_{\text{crit}}}$ increases to 1.3. As ϵ tends to zero, r approaches zero as well and $\frac{L^*}{L_{\text{crit}}} \to 2$.

Window size if $\ell_{\textbf{triple}} \gg \ell_{\textbf{interleaved}}$

We now suppose the single longest triple repeat dominates the lower bound and estimate the size of the critical window. In this case $\ell_{\text{triple}} = L_{\text{crit}} - 1$ is the length of the longest triple repeat. Since we don't have matching lower and upper bounds for triple repeats, we separately compute the critical window size for each.

We start with the lower bound. For $L > L_{\text{crit}}$, the minimum value of N required in order to bridge the longest triple repeat is given by (18) and repeated here:

$$N_{\text{triples}} = \frac{G}{3} \cdot \frac{\log \epsilon^{-1}}{L - L_{\text{crit}}}\,. \tag{35}$$

As for the interleaved repeats case considered earlier, we let L^* be the value of L at which the curve described by constraint (35) intersects the Lander-Waterman coverage value, i.e. $N_{\text{triple}}(L^*) = N_{\text{LW}}(L^*) := N^*$. This is the minimum read length for which coverage of the sequence suffices for reconstruction.

A similar procedure as leading to (28) gives $L^*/L_{\text{crit}} = 3/(3-x)$ with x defined in (29). One can check that the estimates on x in (32) continue to hold, and we therefore get

$$\frac{L^*}{L_{\text{crit}}} \approx \frac{3(r+1)}{3(r+1)-1}\,. \tag{36}$$

For the same example as before, $G \sim 10^8$, $L_{\text{crit}} \sim 1000$, and $\epsilon = 5\%$, we get $r \approx 4.6$ and

$$\frac{L^*}{L_{\text{crit}}} = \frac{3(r+1)}{3(r+1)-1} \approx 1.06\,.$$

Changing ϵ to 10^{-5} makes $\frac{L^*}{L_{\text{crit}}} \approx 1.17$, and as ϵ (and hence also r) tends to zero, $\frac{L^*}{L_{\text{crit}}} \to \frac{3}{2}$.

The analogous computation for L^*/L_{crit} for the upper bound, as given by N_3^{all} in (18), yields

$$\frac{L^*}{L_{\text{crit}}} = \frac{r+1}{r + \frac{\log 3}{\log \epsilon^{-1}}} \approx 1.12\,, \tag{37}$$

for the example with $G \sim 10^8$, $L_{\text{crit}} \sim 1000$, and $\epsilon = 5\%$. The critical window size of the upper bound is about twice as large as that of the lower bound for typical values of G and L_{crit}, with ϵ moderate. But as $\epsilon \to 0$, we see from (37) that $L^*/L_{\text{crit}} \to \infty$, markedly different to the $L^*/L_{\text{crit}} \to \frac{3}{2}$ observed for the lower bound.
